# Adipose Mesenchymal Stem Cells Promote Wound Healing by Modulating Expression of SERPINE1 in Dermal Fibroblasts and Keratinocytes

**DOI:** 10.1155/sci/5541440

**Published:** 2026-01-07

**Authors:** YeHua Liang, Qinqian Sun, Jiaqi Sun, Mingyuan Xu, Jinghong Xu, Yijia Yu

**Affiliations:** ^1^ Department of Plastic Surgery, The First Affiliated Hospital, School of Medicine, Zhejiang University, Hangzhou, Zhejiang, China, zju.edu.cn; ^2^ Zhejiang University School of Medicine, Hangzhou, Zhejiang, China, zju.edu.cn; ^3^ Department of Plastic Surgery, The Third Clinical Medical College of Zhejiang Chinese Medical University, Hangzhou, Zhejiang, China, zcmu.edu.cn

**Keywords:** adipose-derived stem cell, fibroblasts, keratinocytes, SERPINE1, wound healing

## Abstract

**Background:**

Adipose‐derived mesenchymal stem cells (ADSCs) have great potential in the realm of tissue repair and regenerative medicine. However, the exact effects of ADSCs on the healing of skin wounds and the underlying mechanisms remain unexplored. Here, we investigated the effects of ADSCs on fibroblasts and keratinocytes and their related molecular mechanisms in wound healing.

**Methods:**

We used a murine model in vivo and a Transwell coculture system in vitro. The proliferation and migration abilities of human dermal fibroblasts (HDFs) and human immortalized keratinocytes (HaCaT) were analyzed after coculture with ADSCs, and the target molecules were investigated by transcriptome sequencing. We further investigated phenotypic changes by knocking down and overexpressing the target molecule and analyzed the potential mechanisms.

**Results:**

We successfully extracted, expanded, and identified ADSCs. ADSCs not only accelerated wound healing in mice but also improved healing quality. Coculture with ADSCs augmented the proliferation and migration capacities of main skin cells in vitro. RNA sequencing analysis revealed that the level of serpin family E member 1 (SERPINE1) in both HDF and HaCaT was significantly regulated by ADSCs. Knockdown of SERPINE1 restrained the proliferation and migration phenotypes of HDF and HaCaT, while overexpression of SERPINE1 did exactly the opposite. Pathway enrichment analysis revealed that SERPINE1 was mainly related to PI3K‐Akt and MAPK signaling pathways.

**Conclusion:**

The in vivo model and in vitro cell test demonstrate that ADSC effectively promotes cutaneous wound healing by augmenting the proliferation and migration of fibroblasts and keratinocytes through upregulating SERPINE1, which provides novel insights into the biological roles of SERPINE1 in wound healing and suggests ADSC has a promising future in skin injury therapy.

## 1. Introduction

Wound healing is a fundamental and complex process of skin barrier function restoration that consists of a highly sequential, coordinated, and temporally overlapping series of events: hemostasis, inflammation, proliferation, and remodeling [1]. Disruptions of these physiologic processes can lead to the formation of complex wounds. Chronic wounds (CWs) pose a growing health and economic burden to society. In developed countries, the annual management of CWs allocates 6% of total healthcare expenditures. Statistical reports from NIH demonstrated an annual cost of $20 billion for CWs management [2]. Despite the considerable progress involved in wound healing, the specific mechanism of wound healing has not been fully understood, and more effective treatment strategies are needed [3].

During the wound‐healing process, various cells work together to restore the integrity of the skin barrier. Among them, fibroblasts are the most abundant cells in the dermis and play a crucial role in synthesizing and remodeling the extracellular matrix (ECM). The proliferation, differentiation, and migration of fibroblasts are essential for proper wound healing [4]. Keratinocytes are the main components of epidermal cells. Human skin heals exclusively by reepithelialization, which is reliant on the proliferation and subsequent migration into the wound bed of keratinocytes [5]. As such, the regulation of fibroblasts and keratinocytes is a vital target for promoting wound healing.

Cell therapy has received much attention for its applications in skin tissue repair, with adipose‐derived mesenchymal stem cells (ADSCs) being among the most promising stem cell populations due to their abundance, ease of isolation, low immunogenicity, and versatility [6]. During wound healing, their interactions with skin cells are involved in regulating skin homeostasis. ADSCs can secrete a rich secretome including cytokines, growth factors, and chemokines, whereby cell proliferation and differentiation, migration, and an improvement in the cellular and microenvironment protection occurred [6–10]. In addition, ADSCs have been shown to accelerate reepithelialization of wounded areas [11]. However, the precise role and underlying mechanisms of ADSCs in wound healing remain largely unknown.

The serine protease/plasmin/matrix metalloproteinase axis plays a pivotal role in stromal remodeling within the wound microenvironment, and strict regulation of protease expression and activity is necessary for proper cell proliferation and migration. Targeting elements within this cascade may lead to new therapies for fibrotic diseases and CWs [12]. Indeed, SERPINs are crucial injury‐response factors as they participate in virtually all stages of repair where they regulate coagulation, fibrinolysis, inflammation, myofibroblast differentiation, and stromal remodeling [12]. One such gene, serine protease inhibitor clade E member 1 (SERPINE1 or PAI‐1), is upregulated in epidermal wounds in response to injury. SERPINE1 is originally recognized for its regulation of fibrinolysis [12, 13] but has also been implicated in reepithelialization of human skin wounds [14]. SERPINE1 is upregulated in several cell types during injury repair and helps regulate collagen remodeling, cell migration, and proliferation [12]. Elevated levels of SERPINE1 during wound healing inhibit uPA/tPA/plasmin and plasmin‐dependent MMP activities, which expedite wound healing [15]. Thus, control (CON) of SEPRINE1 expression and activity is for optimal wound repair outcomes.

In this study, we aimed to uncover the effects of ADSCs on wound healing in murine models in vivo. Furthermore, we delved into the intricate relationship between ADSCs and two critical cells in the wound healing cascade, human dermal fibroblasts (HDFs) and human immortalized keratinocytes (HaCaT), using a transwell coculture system in vitro to elucidate their biological functions and underlying molecular mechanisms. Our findings provide novel insights into wound healing therapies.

## 2. Materials and Methods

### 2.1. Isolation of Human Adipose Mesenchymal Stem Cells

Human adipose tissues harvested from tissues discarded after liposuction of healthy female patients at the Department of Plastic Surgery, the First Affiliated Hospital of Zhejiang University. Each donor provided written informed consent. The research protocol was approved by the Clinical Research Ethics Committee of the First Affiliated Hospital, College of Medicine, Zhejiang University (2020IIT943). Adipose tissues were washed 2–3 times with sterile saline and then digested by 1% collagenase type I (Solarbio, China) at 37°C for 30 min with intermittent shaking. Digestion was terminated by complete DMEM/F12 medium (Servicebio, China), followed by centrifugation at 1000 rpm for 5 min. Discard the upper layer of the solution to obtain cell sediments and then resuspend in a complete medium containing DMEM/F12, 10% FBS (Gibco, USA), and 100 U/mL penicillin–streptomycin (Servicebio, China). The primary ADSCs (defined as passage 0) were cultured in 5% CO_2_ at 37°C in an incubator for 3–5 days until reaching 70%–80% confluence. Cells were harvested by 0.25% trypsin‐EDTA (Gibco, USA), then diluted 1:2–1:3 and plated for subculture. ADSCs at passages 3–7 were used in experiments.

### 2.2. Characterization of ADSCs

Third‐passaged ADSCs were harvested, and a total of 1 × 10^6^ cells were treated with the fluorescence‐conjugated antibodies (all from Biolegend, USA), including PE‐anti‐human CD44, PE‐anti‐human CD90, PE‐anti‐human CD73, PE‐anti‐human CD45, PE‐anti‐human CD31, and phosphate‐buffered saline (PBS; Servicebio, China) was used as a CON. Antibodies were added to centrifuge tubes (1 μL each) and incubated at room temperature in the dark for 30 min. After centrifugation at 1000 rpm for 3 min, the samples were resuspended in 500 μL PBS for flow cytometry (BD LSR II, USA), and the data were analyzed with FlowJo 7.6.1 software (TreeStar Inc., San Carlos, CA, USA).

Adipogenic differentiation was performed according to the OriCell Kit (Cyagen, China, HUXMD‐90031). Briefly, ADSCs were inoculated in six‐well plates at 2 × 10^4^/well until the confluence reached 100%. Adipogenic differentiation was performed using basic medium A (containing FBS, penicillin–streptomycin, glutamine, insulin, 3‐isobutyl‐1‐methyl xanthine, rosiglitazone, and dexamethasone) for 3 days and basic medium B (containing FBS, penicillin–streptomycin, glutamine, and insulin) for 1 day, and they alternated 4 times. Osteogenic differentiation was performed using basic medium (containing FBS, penicillin–streptomycin, glutamine, ascorbate, β‐glycerophosphate, and dexamethasone) for 3 weeks (Cyagen, China, HUXMD‐90021). After induction, 4% paraformaldehyde (Servicebio, China) was used to immobilize the cells for 30 min, and oil red O and alizarin red S staining were used to detect the intracellular accumulation of lipid vacuoles and the formation of bone matrix, respectively. Staining was observed and imaged under a microscope (Olympus Corporation, Japan).

Chondrogenic differentiation was performed according to the OriCell Kit (Cyagen, China, HUXMD‐90041). ADSCs were harvested and resuspended in 15 mL centrifuge tubes at 4 × 10^5^/tube. The ADSCs were incubated in basal medium containing ascorbate, dexamethasone, insulin ferro‐selenium transporter supplement, sodium pyruvate, proline, and transforming growth factor‐β3, and we replaced the medium every 3 days. Chondrogenic induction was continued until the formation of chondrogenic pellets with diameters ranging from 1.5 to 2.0 mm. The cartilage balls were fixed with 4% paraformaldehyde and finally stained with Alcian blue. Staining was observed and imaged under a microscope (Olympus Corporation, Japan).

### 2.3. In Vivo Experimental Design

Animal experiments complied with ARRIVE guidelines 2.0 and were approved by the Tab of Animal Experimental Ethical Inspection of the First Affiliated Hospital, College of Medicine, Zhejiang University. Female 6–8 weeks BALB/c nude mice weighing ~ 20 g (*n* = 8) were obtained from the Vital River Laboratory Animal Technology Co., Ltd (Beijing, China) and maintained in SPF conditions (the First Affiliated Hospital of Zhejiang University Laboratory Animal Center). Keep mice in standard conditions of 21°C and a 12‐h light–dark cycle with free access to food and water. The animals were anesthetized with intraperitoneal injections of ketamine (100 mg/kg, Vetnil). As reported in a previous study [16], two dorsal 6‐mm punch‐out full‐skin thickness wounds were inflicted on either side of the mouse’s midline using a sterile biopsy punch. A full‐thickness wound extending through the panniculus carnosus was made using an iris scissor. Immediately after wounding, 200 μL PBS with 1 × 10^6^ ADSCs (treatment group) or 200 μL PBS only (CON group) was injected at four different sites centered on the periphery of the wound in the shape of a “+”, using a 25G needle for subcutaneous injection. With each mouse acting as its CON, animal numbers were minimized according to the “power analysis” tool and previous study [17] (*n* = 4 per group, repeated twice, *n* = 8 in total). Finally, the mice were individually housed.

Any progressive weight loss or observable behavioral changes, such as decreased motor activity, altered eating patterns, vocalization, self‐mutilation, or piloerection, were considered humane endpoints for euthanasia and led to animal exclusion from the study. No animals were excluded from the study.

### 2.4. Wound Healing Assessment

The wounds were photographed on days 0, 1, 3, 5, 7, and 9 after wounding. Afterward, the size of the wound beds was measured using ImageJ software and calculated as follows: wound bed size = (actual wound area/original wound area) × 100%. The wound assessment was conducted in a blinded manner.

### 2.5. Histology and Immunohistochemistry Analysis

The skin at the wound site of each group was cut on day 9 and fixed overnight with 10% buffered formalin at room temperature. The sections were stained with hematoxylin and eosin (H&E) and Masson’s trichrome (MT). Immunohistochemistry was performed with the use of formalin‐fixed and deparaffinized sections. Primary antibodies against proliferating cell nuclear antigen (PCNA) and alpha smooth muscle actin (α‐SMA) (Abcam, UK) were incubated overnight at 4°C. After washing off the unbound primary antibody, secondary antibodies were incubated for 60 min at room temperature. Diaminobenzidine was used for color development. The sections were observed under a light microscope. For quantitative analysis of IHC staining, five random fields of view per sample were captured under a high‐power microscope (e.g., 400x magnification). Image analysis was performed using ImageJ software (National Institutes of Health, USA). The percentage of positive cells was calculated by dividing the number of cells with positive nuclear (for PCNA) or cytoplasmic (for α‐SMA) staining by the total number of cells in each field. The integrated optical density (IOD) of the positive staining was also measured. Data are presented as mean ± SD from three independent experiments. All the slides were evaluated by a veterinary pathologist in a blinded manner.

### 2.6. Coculture Condition

To evaluate the effect of the soluble factors secreted by ADSCs on HDFs and HaCaT, we assembled a two‐chamber coculture system permitting the exchange of soluble diffusible factors without direct cell‐to‐cell contact. ADSCs (5 × 104 cells/well) were placed in the upper compartment of the transwell insert (0.45 μm pores; Corning Costar, USA), while HDFs or HaCaT (5 × 10^4^ cells/well) were added to the lower chambers containing DMEM with 10% FBS. The CON group had only medium added to the upper chamber. After coculturing for 24 h, HDFs and HaCaT were harvested for further analysis.

### 2.7. Cell Proliferation Assay

Cell proliferation capacity was evaluated using the cell counting Kit‐8 (CCK‐8) assay (APE×BIO Technology LLC, Houston, USA). The cell suspensions (5 × 10^3^/well, 100 μL) were seeded into 96‐well plates and incubated for 24, 48, 72, and 96 h. Then, 10 μL of CCK‐8 solution was added to each well and incubated at 37°C for 4 h. The optical value was measured at an absorbance of 450 nm using a spectrophotometer.

### 2.8. Cell Scratch Assay

Migration properties were assessed using the scratch assay. Cells were first seeded into silicone culture inserts (Ibidi, Germany). The culture inserts were removed after the cells had adhered to the bottom. The wound area was photographed and measured with ImageJ (National Institutes of Health, Bethesda, MD, USA) at 0 and 24 h after the culture inserts were removed.

### 2.9. Transwell Migration Assay

For migration assays, HDFs or HaCaT were resuspended in serum‐free DMEM and deposited onto an 8‐mm‐pore‐size Transwell chamber (BD Falcon, USA). The lower chamber was filled with 800 μL complete medium. After 48 h of incubation, cells on the upper surface of the membrane were removed with a cotton swab. Cells adhering to the lower membrane were fixed with the fixative solution and then stained with a staining solution of Wright–Giemsa Stain Kit (Nanjing Jiancheng Institute of Bioengineering, China). Images were taken with a phase contrast microscope (Olympus, Japan).

### 2.10. RNA Sequencing

Total RNA was isolated using the RNAiso Plus kit (Vazyme Biotech Co., Ltd, Nanjing, China), and mRNA was enriched using magnetic beads with oligo (dT). The mRNA was converted into individual cDNA libraries following fragmentation. These libraries underwent quality CON checks, being sequenced on the Illumina NovaSeq 6000 platform. Differential expression analysis was performed with a significance threshold set at padj <0.05 and |log2foldchange| > 1.0.

### 2.11. Quantitative Real‐Time PCR (qRT‐PCR) Analysis

Total RNA was isolated using the TRIzol reagent (Invitrogen, USA). The RNA was reverse transcribed into cDNA using the PrimeScript RT kit (TaKaRa, Japan). The expression of mRNA was measured using qRT‐PCR with SYBR Green Kit (TaKaRa, Japan) on a CFX96TM Real‐Time System (BioRad, USA). Expression levels were analyzed using the 2^−△△Ct^ method and normalized to GAPDH. The primer sequences are listed in Table 1.

**Table 1:** Sequences of primers used for qRT‐PCR.

Target gene	Forward primer 5′–3′	Reverse primer 3′–5′
*BIRC3*	CCTGAGCATGCAGACACACGCAGCCCGT	CCCAGCACCTCAGCCCACCATCACAGCA
*CCL20*	AGGCAGAAGCAGCAAGCAACTACGACTGT	ACCCAGTTCTGCTTTGGATCAGCGCACAC
*CXCL3*	CCCCTCCCACCTGCCGGCTCC	GGGGTCCTGGGGGCGTCACCG
*TNFAIP3*	CCCCACCCACAGCACCCAGCCTT	TCTGCCGGGCGTGGCAACGCTCA
*CXCL5*	TGGCGCCGCTGGCATTTCTGTTGCTG	TTCCACCGTAGGGCACTGTGGACCTGCA
*SERPINE1*	AGCGGGACCTAGAGCTGGTCCAGGGCTT	TCACCAGCACCAGGCGTGTCAGCTCGTC
*CYP24A1*	TGACCCCCGTGGAGCTGCACAAGC	AGGAAATCCGCACCAGGCTGCTGGGAA
*GAPDH*	ACCATGGAGAAGGCCGGGGCCCAC	TGACCTTGGCCAGGGGGGCTAAGCA

### 2.12. Transfection

Small interfering RNA (siRNA) for SERPINE1 (si‐SERPINE1) and CON siRNA (si‐NC) were synthesized by GenePharma (Shanghai, China). All cell transfections were performed using Lipofectamine RNAiMAX transfection reagent (Invitrogen, USA). To overexpress (OE) SERPINE1, cells were cultivated overnight in 24‐well plates, and lentiviral infection was introduced (sourced from GenePharma, China). Stable transfectants were selected using 2 μg/mL puromycin screening and verified by qRT‐PCR.

### 2.13. Western Blot

Western blot analysis was performed using standard protocols. Briefly, cells were lysed with RIPA lysis buffer on ice, and the protein concentration was measured using a BCA Protein Assay Kit (Servicebio, China). Equal amounts of protein were separated by SDS–PAGE and transferred onto PVDF membranes (Millipore, USA). The membranes were blocked with 5% skim milk in TBST for 1 h at room temperature and then incubated overnight at 4°C with the following primary antibodies: anti‐serpin E1/PAI‐1 (1:1000, Rabbit polyclonal), anti‐α‐SMA (1:1000, Rabbit polyclonal), anti‐PCNA (1:5000, Rabbit polyclonal), anti‐GAPDH (1:5000, Rabbit polyclonal) (all from Proteintech, China), and antibeta‐tubulin (1:2000, Mouse Recombinant) (Servicebio, China). After washing, the membranes were incubated with corresponding HRP‐conjugated secondary antibodies (1:3000, Proteintech) for 1 h at room temperature. Protein bands were visualized using an ECL detection system (Monad QuickChemi GD50202, China), and band intensities were quantified with ImageJ software, normalized to GAPDH or β‐Tubulin as a loading CON.

## 3. Statistical Analyses

All data are presented as mean ± standard error of the mean (SEM). Each experiment was conducted in triplicate at least. Statistical differences between two groups were compared using Student’s *t*‐test, and those among more than two groups were compared using a one‐way or two‐way ANOVA. A value of *p* < 0.05 was considered significant. All statistical analyses were performed using Prism 9 software (CA, USA).

## 4. Results

### 4.1. Characterization of ADSCs

ADSCs had a typical fibroblastic morphology under a light microscope, with elongated spindle or polygonal shapes, ample cytoplasm, and oval nuclei. They displayed parallel, radiating, or whirlpool‐like growth patterns with distinct cell boundaries, conforming to the characteristic growth patterns of ADSCs (Figure 1A,B). The cells demonstrated multipotent differentiation capacity, evidenced by adipogenic differentiation visualized through oil red O staining of lipid droplets in adipocytes (Figure 1C). Osteogenic differentiation was confirmed with alizarin red S staining of calcium deposits in osteoblasts (Figure 1D). Chondrogenic differentiation was revealed by alician blue staining of cartilage matrices (Figure 1E). Flow cytometric analysis of ADSCs showed remarkably positive for the stem cell‐specific markers CD44, CD73, and CD90, and negative expression of the hematopoietic markers CD31 and CD45 (Figure 1F), indicating successful isolation of ADSCs.

Figure 1Isolation and characterization of ADSCs. (A) Observation of P1 generation ADSCs under an inverted microscope, scale bar: 400 μm; (B) P1 ADSCs display characteristic fibroblastic morphology, scale bar: 200 μm; (C) adipogenic differentiation of ADSCs assessed by oil red O staining, scale bar: 200 μm; (D) osteogenic differentiation ADSCs visualized through alizarin red staining, scale bar: 200 μm; (E) chondrogenic differentiation of ADSCs indicated by alician blue staining, scale bar: 50 μm; and (F) flow cytometry of ADSC surface markers showing positivity for CD90, CD73, and CD44, and negativity for CD31 and CD45.

**Figure:**
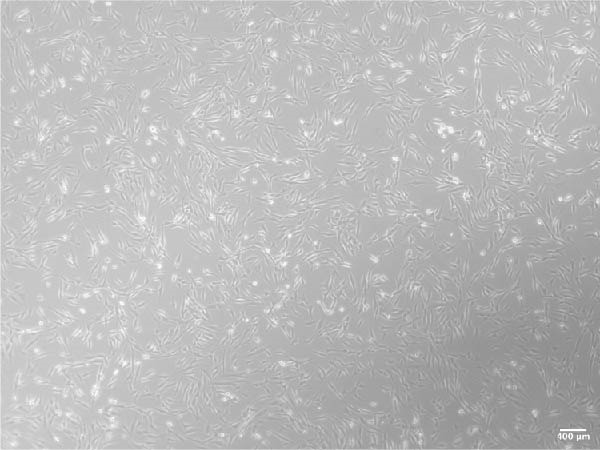
(A)

**Figure:**
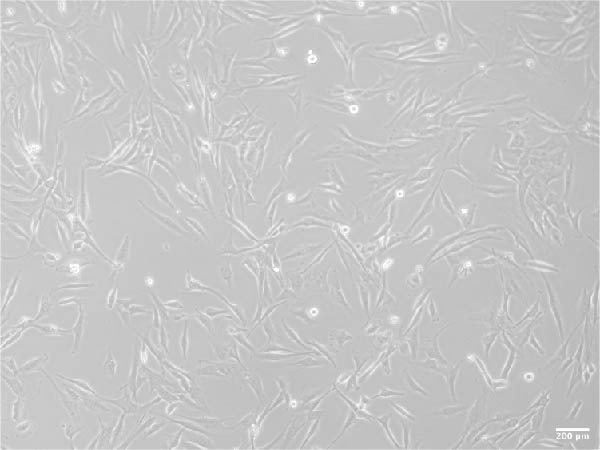
(B)

**Figure:**
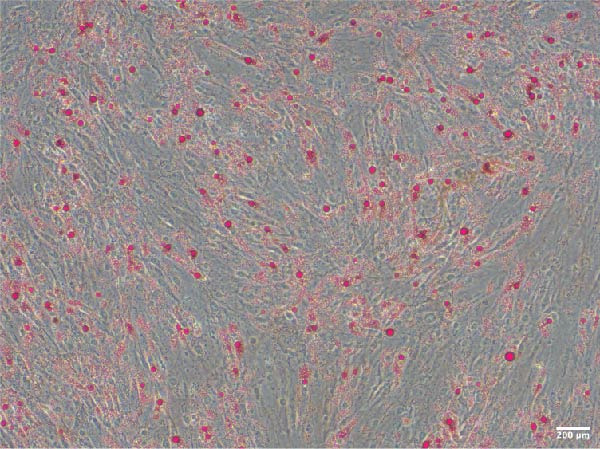
(C)

**Figure:**
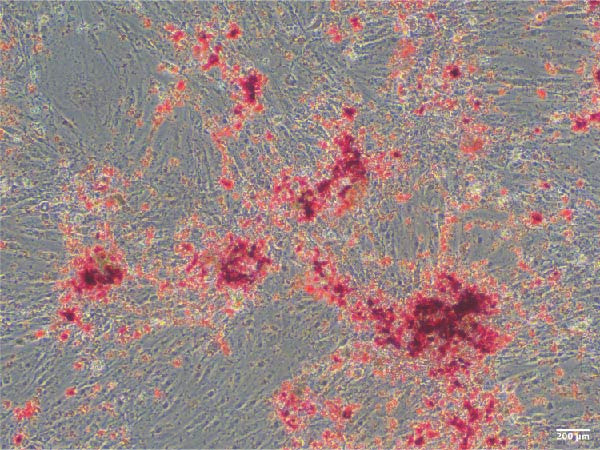
(D)

**Figure:**
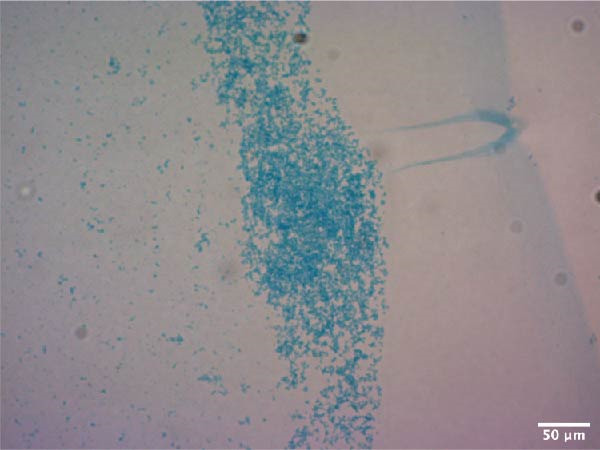
(E)

**Figure:**
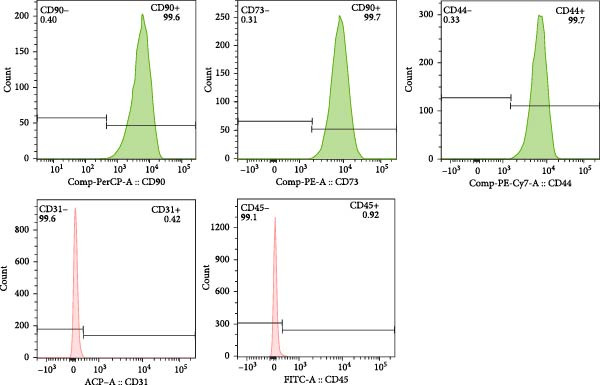
(F)

### 4.2. ADSCs Enhance Cutaneous Wound Healing in Mice

To determine the effect of ADSCs on cutaneous wound healing in vivo, we apply the mouse model. The full‐thickness cutaneous wounds were created on the dorsal skin areas of mice (Figure 2A), followed by the treatment of ADSC (+ADSC, *n* = 8) or without ADSC (PBS, *n* = 8), and there were no exclusions. Wound healing was monitored over 9 days, and digital images of the wound size were taken at indicated times. The ADSC group appeared to have a significantly better velocity of the wound healing process than the CON group, especially on day 9 postwounding (*p* < 0.5; Figure 2B,C).

Figure 2Wound healing potential of ADSC in full‐skin wound injury mice model. (A) Experimental design for wound model preparation. (B) Quantification of residual wound areas relative to the initial wound areas. (C) Representative pictures of wounds on days 1, 3, 5, 7, and 9. Scale bar = 1mm. (D) H&E staining of wounded skin sections in different groups on day 9 postwounding. (E) Masson staining of wounded skin sections in different groups on day 9 postwounding. (F) PCNA immunohistochemical staining in the wounded skin sections on day 9 postwounding. (G) Quantification of PCNA‐positive areas of IHC staining from two groups. (H) α‐SMA immunohistochemical staining in the wounded skin sections on day 9 postwounding. (I) Quantification of α‐SMA‐positive areas of IHC staining from two groups. Results are presented as mean ± SD (*n* = 8 for each group).  ^∗^ *p* < 0.05,  ^∗∗^ *p* < 0.01, and  ^∗∗∗^ *p* < 0.001 vs. control group.

**Figure:**
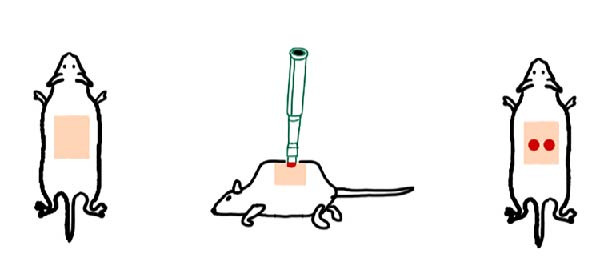
(A)

**Figure:**
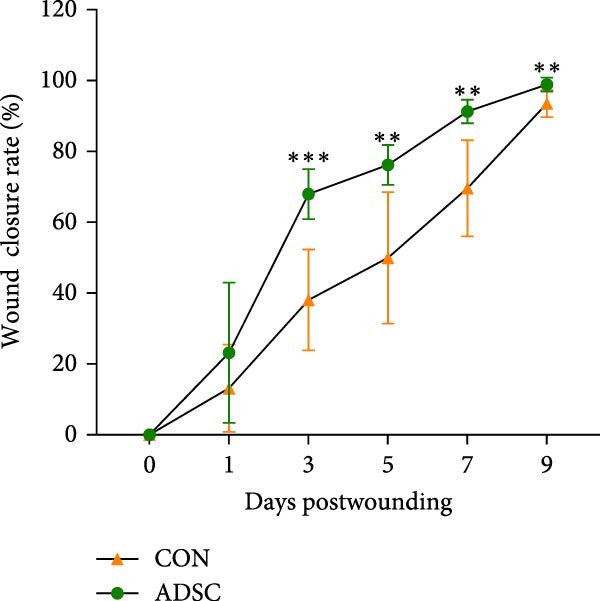
(B)

**Figure:**
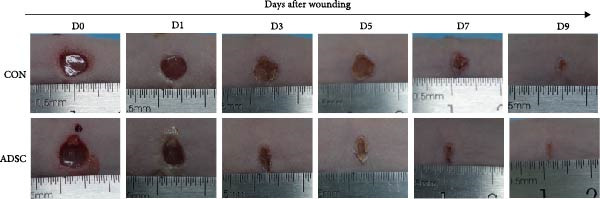
(C)

**Figure:**
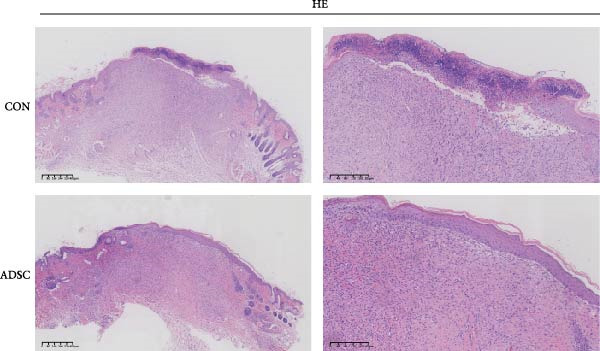
(D)

**Figure:**
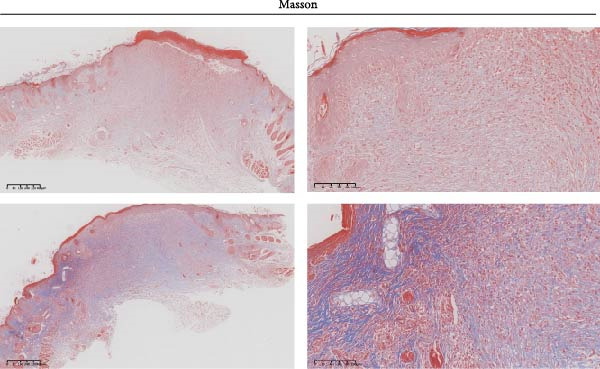
(E)

**Figure:**
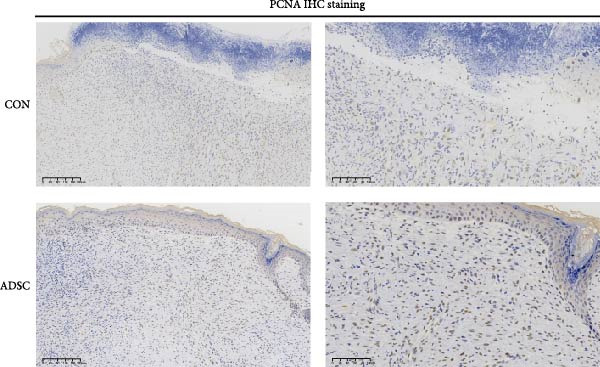
(F)

**Figure:**
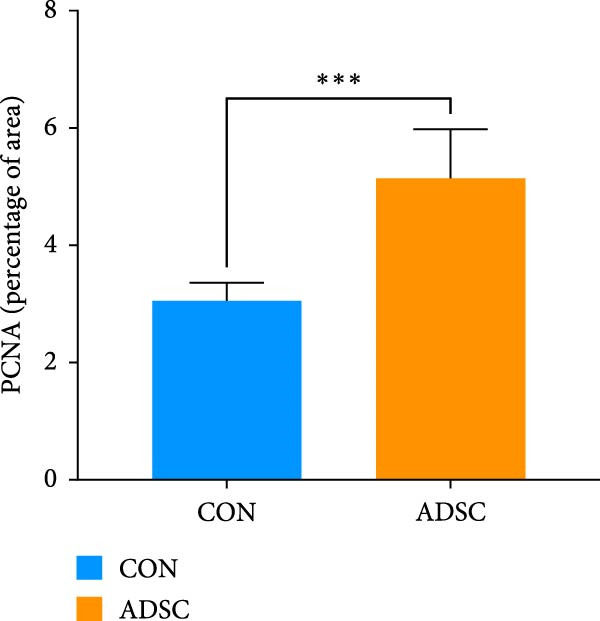
(G)

**Figure:**
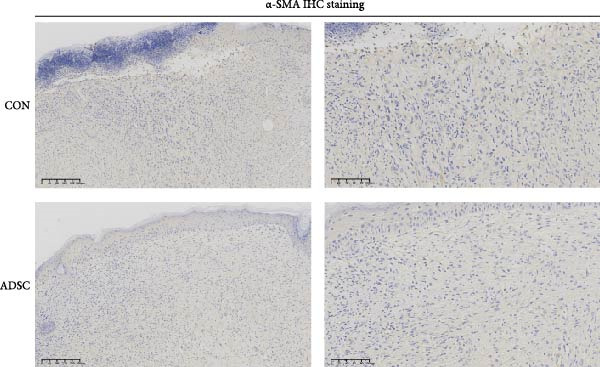
(H)

**Figure:**
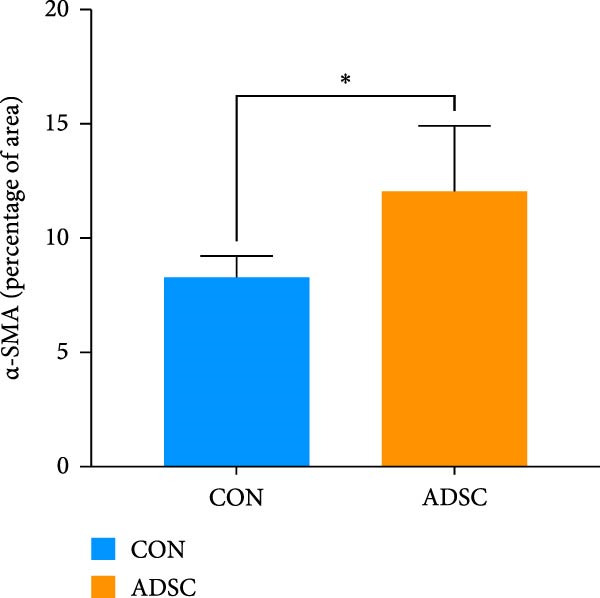
(I)

H&E staining revealed that compared to the PBS group, the ADSCs‐treated group exhibited a more prominent trend of reepithelialization. The continuity of the epithelium was significantly more intact, with a thicker regenerated epidermal layer. Additionally, there was less infiltration of inflammatory cells, the epithelial structure was clearer, and there was enhanced deposition of collagen fibers as well as a more pronounced formation of new blood vessels (Figure 2E). MT staining demonstrated that relative to the PBS group, the ADSCs group had a richer abundance of collagen fibers, which were arranged more regularly and distributed more evenly (Figure 2F).

We also performed immunohistochemical staining for PCNA and α‐SMA. PCNA staining can evaluate the formation of granulation tissue and thus determine wound healing [18]. Immunohistochemical staining of PCNA showed strong positivity in the ADSCs‐treated group (*p* < 0.001), implying increased expression (Figure 2F,G). α‐SMA is a critical marker of myofibroblasts [19], and its presence is widely regarded as a sign of skin healing [20]. The results showed that α‐SMA was highly expressed in the ADSCs group compared with those in the PBS group (*p* < 0.05) (Figure 2H,I). What is more, expression of α‐SMA both at and adjacent to the wound edge indicated the uniform and organized distribution of myofibroblasts in the wound site (Figure 2H). Therefore, we concluded that ADSCs not only accelerate the rate of wound healing in nude mice but also notably enhance the quality of wound healing, underscoring the therapeutic potential of ADSCs in wound regeneration and repair.

### 4.3. ADSCs Promote the Proliferation and Migration of HDF

To elucidate the effect of ADSCs in vitro, we first verified the effects of secreted factors from ADSCs on fibroblasts by culturing cells in two‐chamber dishes and preventing direct contact but permitting the exchange of soluble diffusible factors. After coculturing for 24 h, cell viability was assessed by the CCK‐8 assay. The results revealed that OD values of HDFs on day 3 and day 4 postcoculture were significantly higher compared to the CON group (*p* < 0.05), indicating enhanced proliferative capacity of HDFs (Figure 3A). In the cell scratch assay, the migration rate of HDFs cocultured with ADSCs was significantly increased relative to the CON, suggesting that ADSCs facilitate HDF migration (Figure 3B). Subsequently, a significantly greater number of HDFs after coculture migrated through the chambers in the transwell assay, further implying that ADSCs promote HDF migration (Figure 3C). To molecularly validate our immunohistochemical observations in vivo, we examined the protein expression of PCNA and α‐SMA in HDFs following ADSC coculture in vitro. Western blot analysis revealed that coculturing with ADSCs for 24 h significantly upregulated the protein levels of both PCNA and α‐SMA in HDFs compared to the CON group (Figure 3D). These in vitro results directly corroborate the in vivo data, providing robust molecular evidence for the ADSC‐induced enhancement of proliferation and myofibroblast activation. Collectively, these experiments demonstrate that ADSCs enhance both the proliferation and migration of HDFs.

Figure 3ADSCs promote the migration and proliferation of HDF. (A) Cell scratch assay images displaying the dynamic change in HDF migration capacity over 24 h (0–24 h) (200x magnification). (B) Representative images from transwell assays depicting the variation in HDF migratory capability (200x magnification). (C) Graph illustrating the trend of relative optical density (OD) value changes in HDFs measured using the CCK8 assay kit. (D) Detection of α‐SMA and PCNA protein expression in HDFs cocultured with ADSCs via western blotting assay. Group A + F denotes HDFs cocultured with ADSCs for 24 h, whereas CON represents control HDFs cultured independently (*n* = 3).  ^∗^ *p* < 0.05,  ^∗∗^ *p* < 0.01,  ^∗∗∗^ *p* < 0.001,  ^∗∗∗∗^ *p* < 0.0001.

**Figure:**
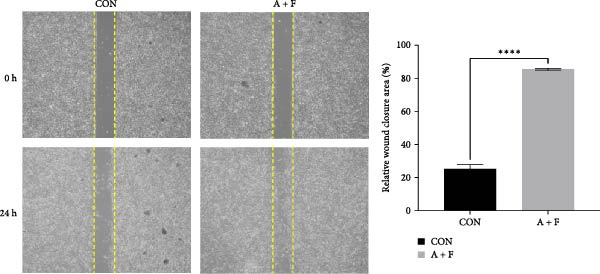
(A)

**Figure:**
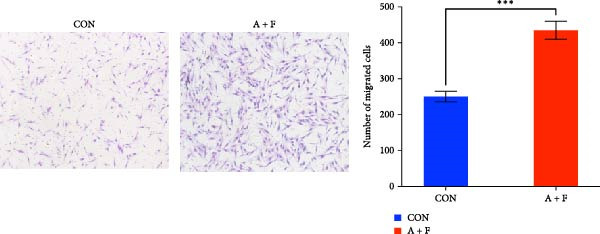
(B)

**Figure:**
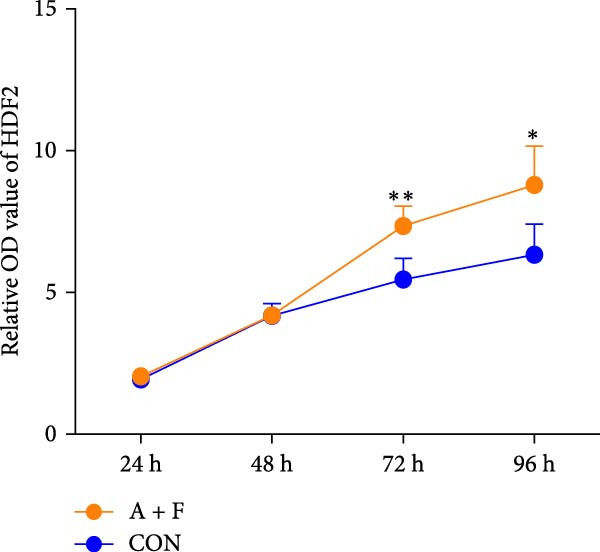
(C)

**Figure:**
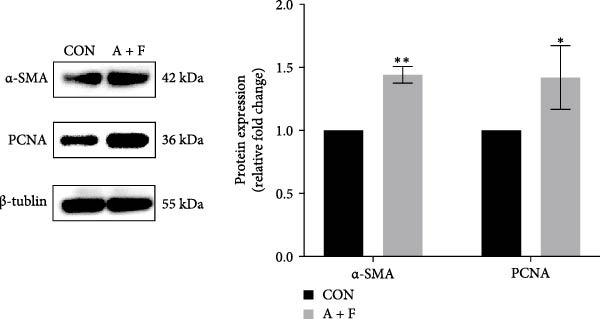
(D)

### 4.4. ADSCs Promote the Proliferation and Migration of HaCaT

To further investigate the effect of ADSCs on epidermal cells, we first verified whether ADSCs were able to exert biological effects on HaCaT. Following 24‐h coculture of ADSCs and HaCaT cells, cell viability assessed via the CCK‐8 assay indicated that the OD values of HaCaT cells on days 2, 3, 4, and 5 postcoculture were significantly higher compared to the CON group, suggesting augmented cellular proliferation capacity of HaCaT cells after coculture with ADSCs (Figure 4A). In the scratch assay, the migration velocity of HaCaT cells under coculture conditions was significantly elevated compared to the CON, implying that ADSCs facilitate HaCaT cell migration (Figure 4B). Moreover, in the transwell assay, a substantial increase in the number of HaCaT cells migrating through the inserts was observed after coculture, further indicating that ADSCs promote HaCaT migration (Figure 4C). Similarly, the proproliferative effect of ADSCs was confirmed in HaCaT cells at the protein level. A significant increase in PCNA expression was detected by Western blot after ADSC coculture (Figure 4D), corroborating the in vivo findings and underscoring the positive impact of ADSCs on keratinocyte proliferation. These findings collectively demonstrate that ADSCs stimulate both proliferation and migration in HaCaT cells.

Figure 4ADSCs promote the migration and proliferation of HaCaT. (A) Cell scratch assay images displaying the dynamic change in HaCaT migration capacity over 24 h (0–24 h) (200x magnification). (B) Representative images from transwell assays depicting the variation in HaCaT migratory capability (200x magnification). (C) Graph illustrating the trend of relative optical density (OD) value changes in HaCaT measured using the CCK8 assay kit. (D) Detection of α‐SMA and PCNA protein expression in HDFs cocultured with ADSCs via western blotting assay. Group A + H denotes HaCaT cocultured with ADSCs for 24 h, whereas CON represents control HaCaT cultured independently (*n* = 3).  ^∗^ *p* < 0.05,  ^∗∗^ *p* < 0.01,  ^∗∗∗^ *p* < 0.001,  ^∗∗∗∗^ *p* < 0.0001.

**Figure:**
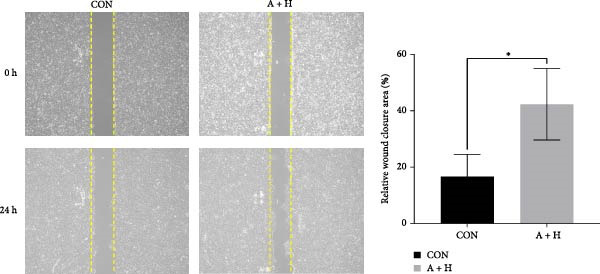
(A)

**Figure:**
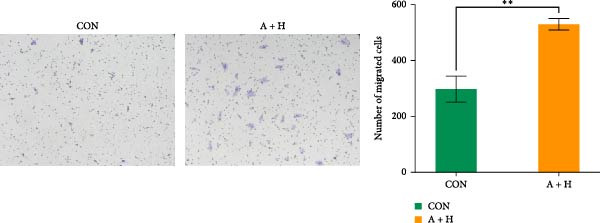
(B)

**Figure:**
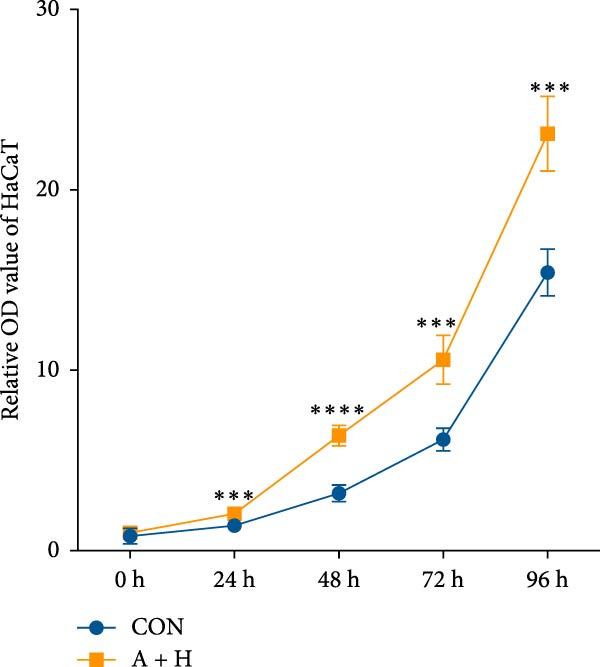
(C)

**Figure:**
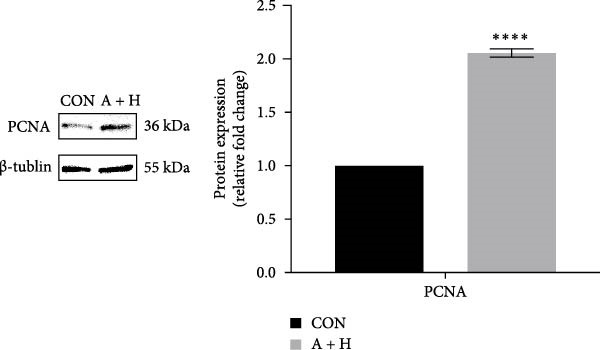
(D)

### 4.5. Transcriptomic Analysis of Differentially Expressed Genes in HDF and HaCaT Cells Under Coculture With ADSCs

We conducted coculture experiments involving ADSCs with HDFs and separately with HaCaT cells, followed by transcriptome sequencing 24 h later. Compared to HDFs cultured alone, cocultured HDFs showed 114 genes significantly downregulated and 10 genes significantly upregulated. Kyoto Encyclopedia of Genes and Genomes (KEGG) pathway analysis revealed notable differences in pathways such as IL‐17, TNF, HIF‐1, and NF‐κB signaling (Figure 5A,C,D,F). HaCaT cells cocultured with ADSCs demonstrated 244 genes significantly downregulated and 58 genes significantly upregulated, with KEGG analysis highlighting significant variations in IL‐17, TNF, and NOD‐like pathways (Figure 5B,C,E,G). To validate the sequencing outcomes, we selected genes with prominent differential expression for verification. Quantitative real‐time polymerase chain reaction (qRT‐PCR) confirmed that, compared to the CON group, cocultured HDFs exhibited significantly increased expression of SERPINE1 and ANGPTL4, alongside reduced expression of INHBA and TNFAIP3 (Figure 5H). Similarly, in cocultured HaCaT cells compared to the CON group, ANGPTL4, CCL20, CXCL8, SERPINE1, and TNFAIP3 expressions were significantly elevated, whereas INSIG1 expression was significantly decreased (Figure 5I). Notably, ANGPTL4 and SERPINE1 showed a marked increase in both cell types. Further, western experiments indicated the expression SERPINE1 protein was upregulated after coculturing with ADSC for 24 h in HDF (Figure 5J) and HaCaT (Figure 5K). Consequently, SERPINE1 was chosen as the target molecule for further study (Figure 5H,I).

Figure 5Transcriptome analysis of differentially expressed genes in HDF and HaCaT cells under coculture with ADSCs. (A) Volcano map of differentially expressed genes identified by RNA‐seq of HDF cells cocultured with ADSCs. (B) Volcano map of differentially expressed genes identified by RNA‐seq of HaCaT cells cocultured with ADSCs. (C) Heat map showing differentially expressed genes in HDF and HaCaT cells treated with ADSCs. (D) Results of GO analysis of HDF cells. (E) Results of GO analysis of HaCaT. (F) Results of KEGG analysis of HDF. (G) Results of KEGG analysis of HaCaT. (H) qRT‐PCR analysis of the mRNA expression levels of differentially expressed ARGs in HDF. (I) qRT‐PCR analysis of the mRNA expression levels of differentially expressed ARGs in HaCaT. (J) Detection of SERPINE1 protein expression by western blot in HDF. (K) Detection of SERPINE1 protein expression by western blot in HaCaT. Data are presented as mean ± SD.  ^∗^ *p* < 0.05,  ^∗∗^ *p* < 0.01,  ^∗∗∗^ *p* < 0.001. GO, Gene Ontology; KEGG, Kyoto Encyclopedia of Genes and Genomes.

**Figure:**
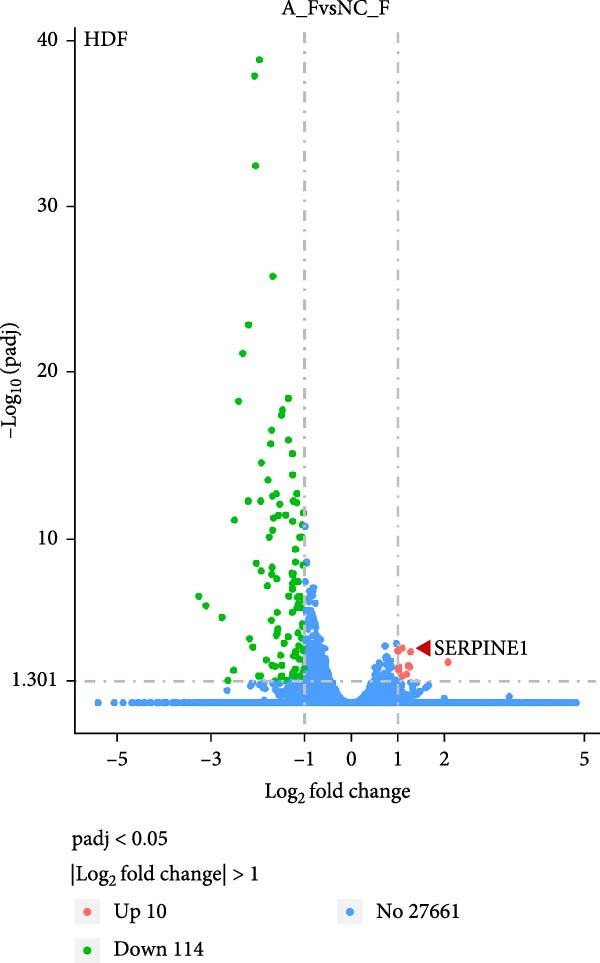
(A)

**Figure:**
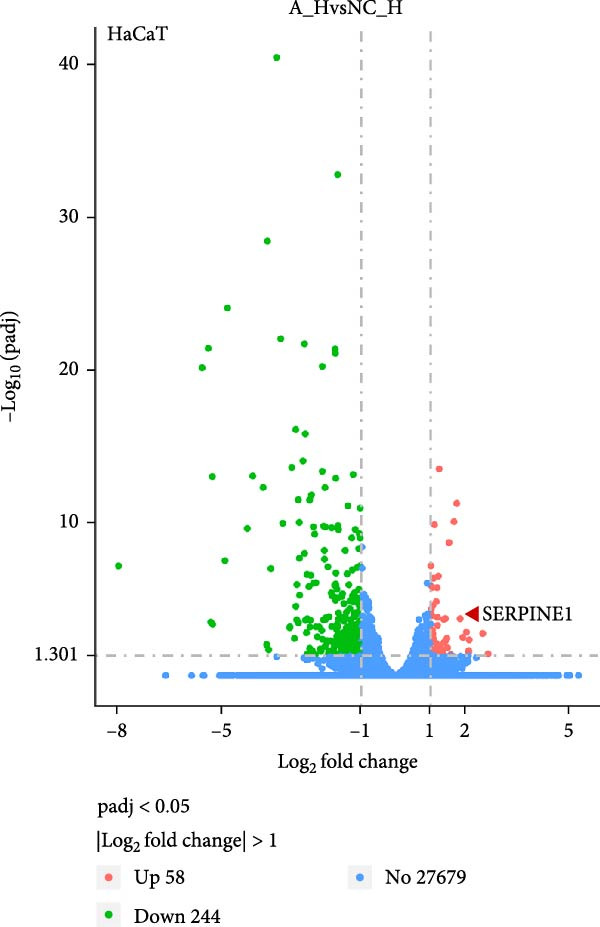
(B)

**Figure:**
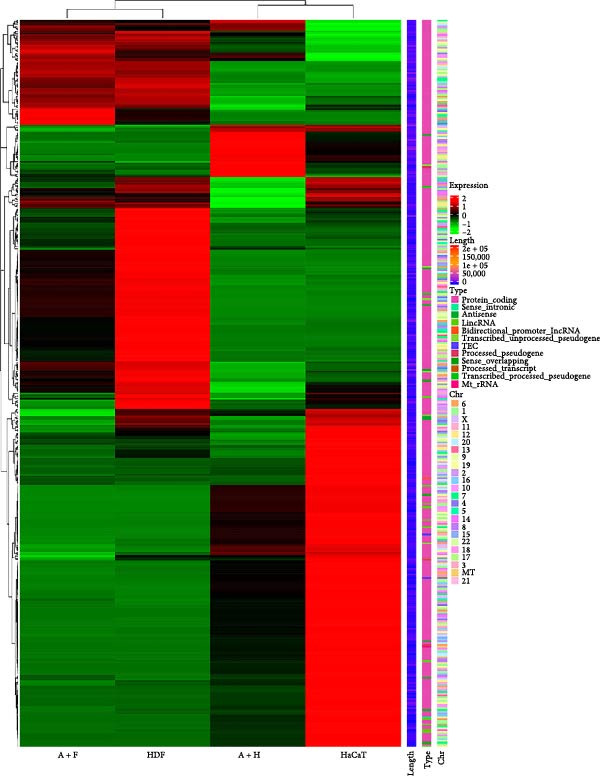
(C)

**Figure:**
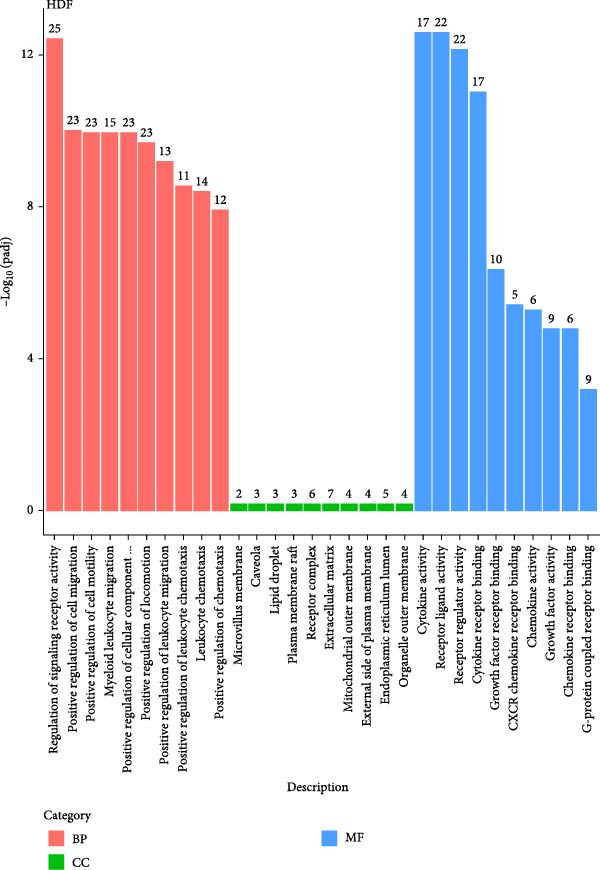
(D)

**Figure:**
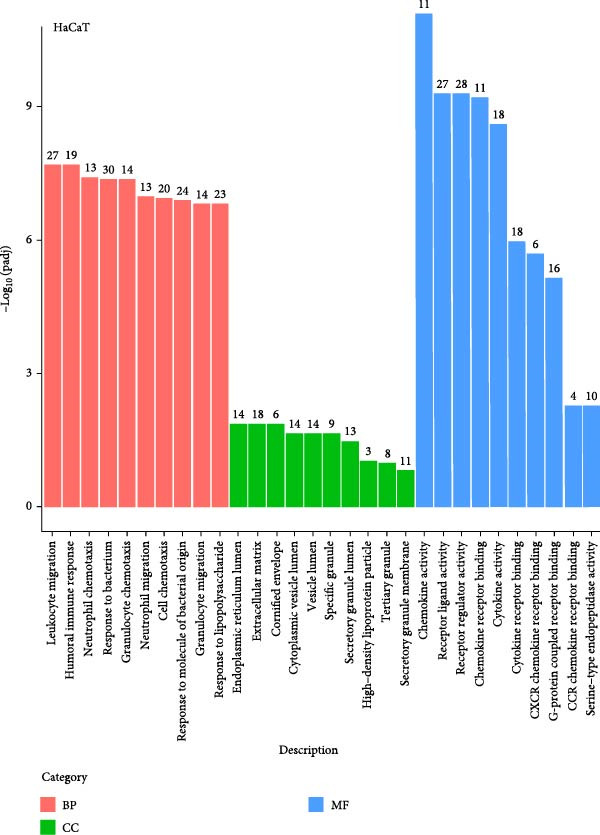
(E)

**Figure:**
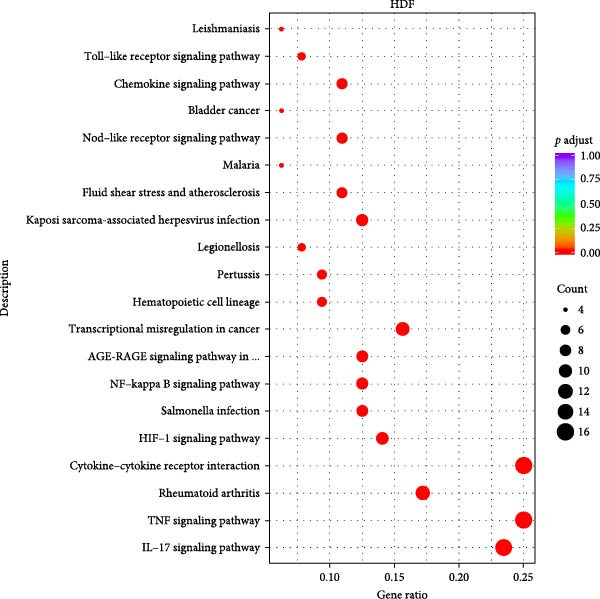
(F)

**Figure:**
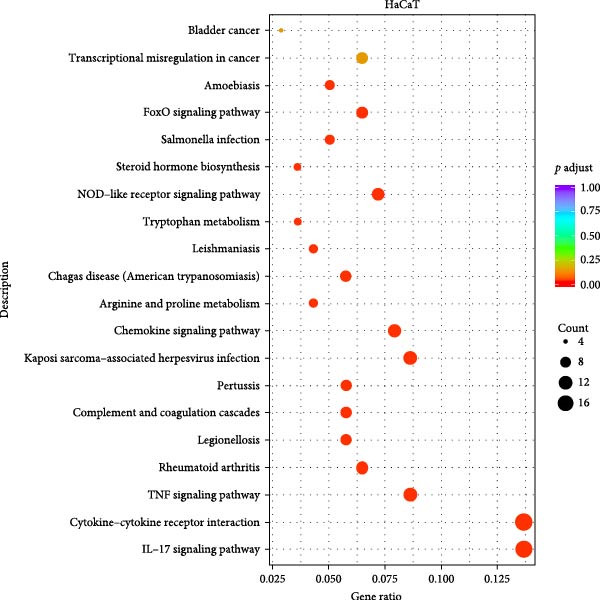
(G)

**Figure:**
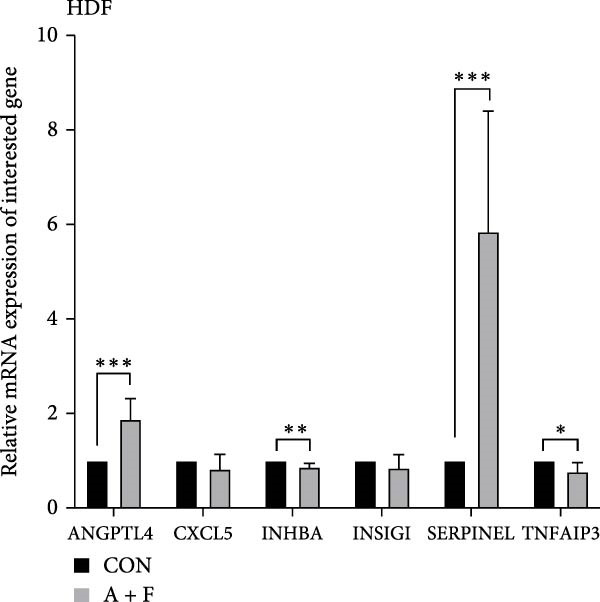
(H)

**Figure:**
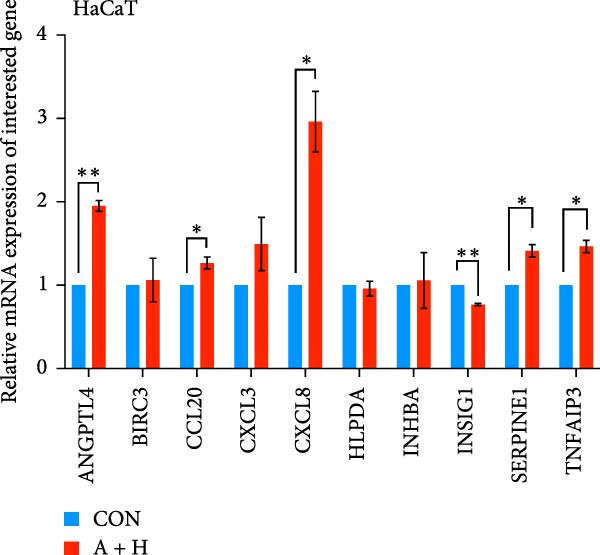
(I)

**Figure:**
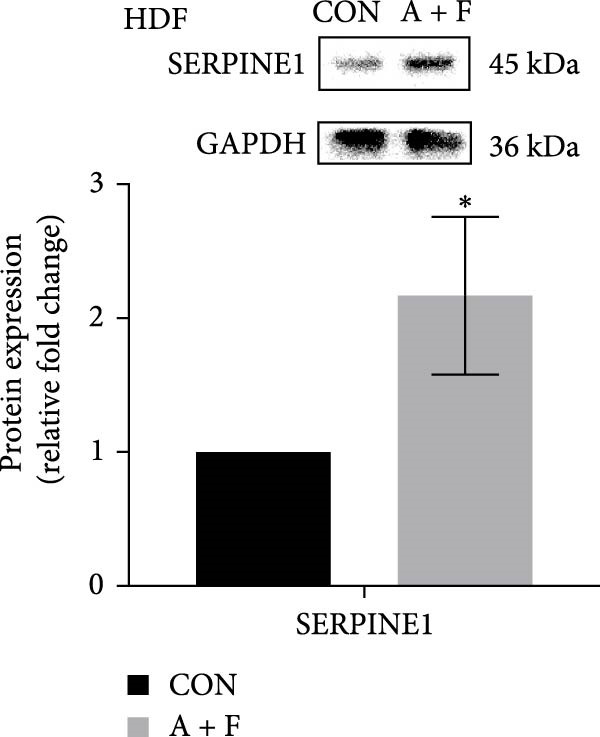
(J)

**Figure:**
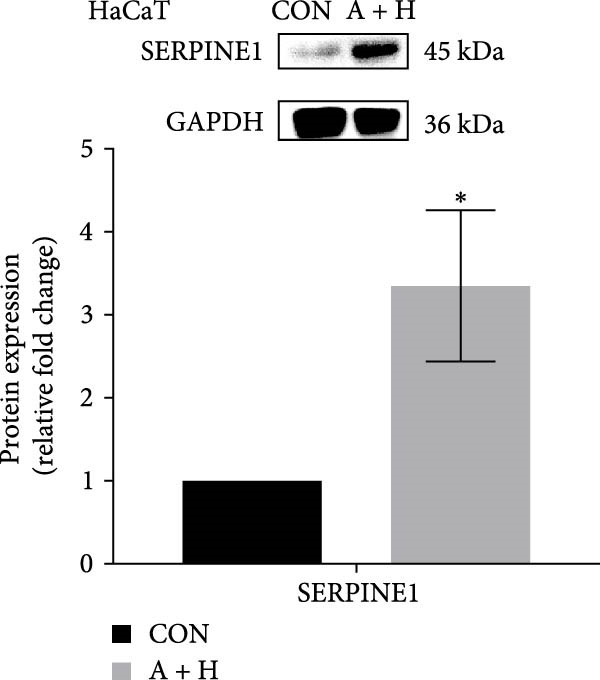
(K)

### 4.6. Phenotypic Validation by Knockdown of SERPINE1 in HDF and HaCaT Cells

To further elucidate the function of SERPINE1, we constructed lentiviruses for SERPINE1 knockdown in HDF and HaCaT using our constructed siRNA and validated the efficiency of knockdown (Figures 6A and 7A).

Figure 6Phenotypic validation by knockdown of SERPINE1 in HDF. (A) mRNA expression profiles in HDFs following knockdown of SERPINE1. KD: SERPINE1 knockdown by si‐SERPINE1; NC (KD): negative control group transduced with si‐NC; CON: untreated control group. (B) Knockdown of SERPINE1 suppressed proliferation in HDFs measured using the CCK‐8 assay. si‐SERPINE1: SERPINE1 knockdown group; NC: negative control group. (C) Cell scratch assay images display a downward trend in si‐SERPINE1 HDFs migration capacity over 24 h (0–24 h) (200x magnification). (D) Transwell cell migration assay showed that the knockdown of SERPINE1 inhibited migration in HDFs (200x magnification) (*n* = 3).  ^∗^ *p* < 0.05,  ^∗∗^ *p* < 0.01,  ^∗∗∗^ *p* < 0.001,  ^∗∗∗∗^ *p* < 0.0001.

**Figure:**
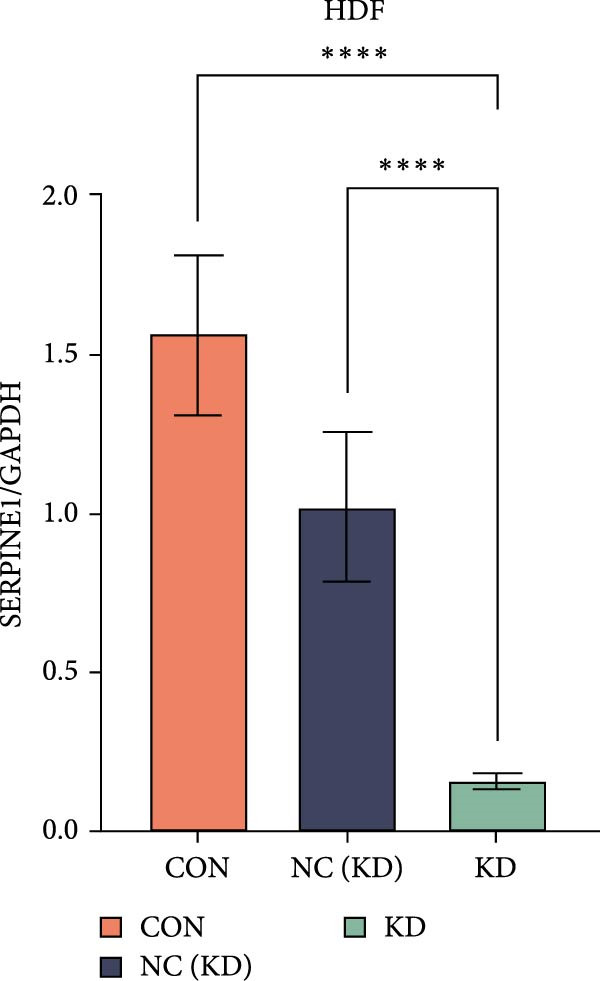
(A)

**Figure:**
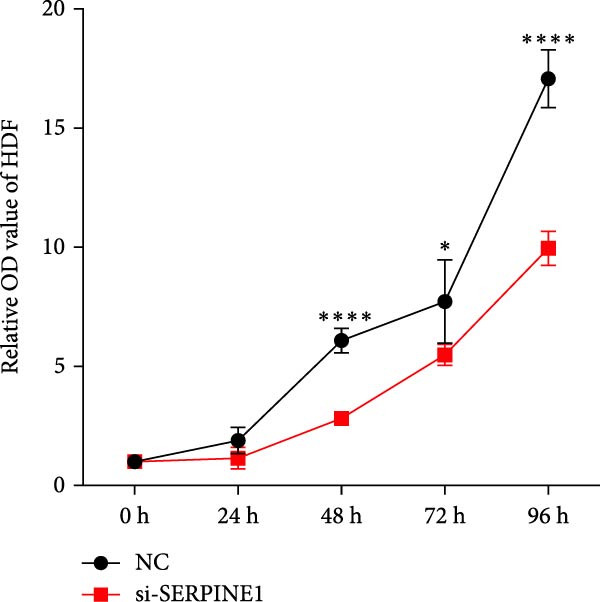
(B)

**Figure:**
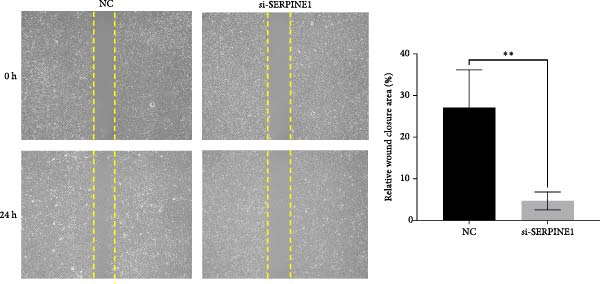
(C)

**Figure:**
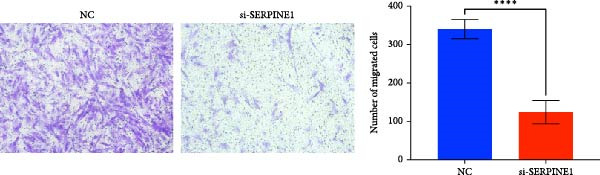
(D)

Figure 7Phenotypic validation by knockdown of SERPINE1 in HaCaT cells. (A) mRNA expression profiles in HaCaT following knockdown of SERPINE1. KD: SERPINE1 knockdown by si‐SERPINE1; NC (KD): negative control group transduced with si‐NC; CON: untreated control group. (B) Knockdown of SERPINE1 suppressed proliferation in HaCaT, measured using the CCK‐8 assay. si‐SERPINE1: SERPINE1 knockdown group; NC: negative control group. (C) Cell scratch assay images display a downward trend in si‐SERPINE1 HaCaT migration capacity over 24 h (0–24 h) (200x magnification). (D) Transwell cell migration assay showed that knockdown of SERPINE1 inhibited migration in HaCaT (200x magnification) (*n* = 3).  ^∗^ *p* < 0.05,  ^∗∗^ *p* < 0.01,  ^∗∗∗^ *p* < 0.001,  ^∗∗∗∗^ *p* < 0.0001.

**Figure:**
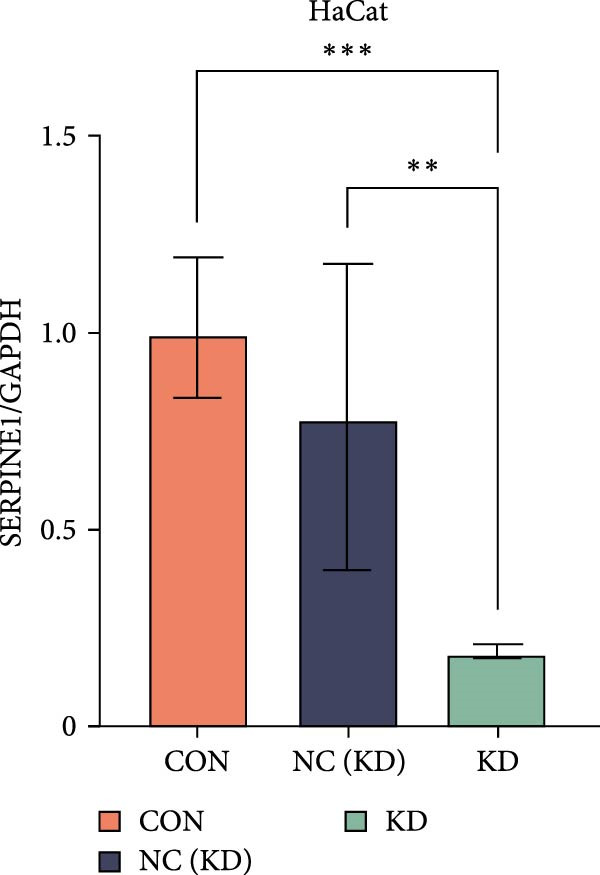
(A)

**Figure:**
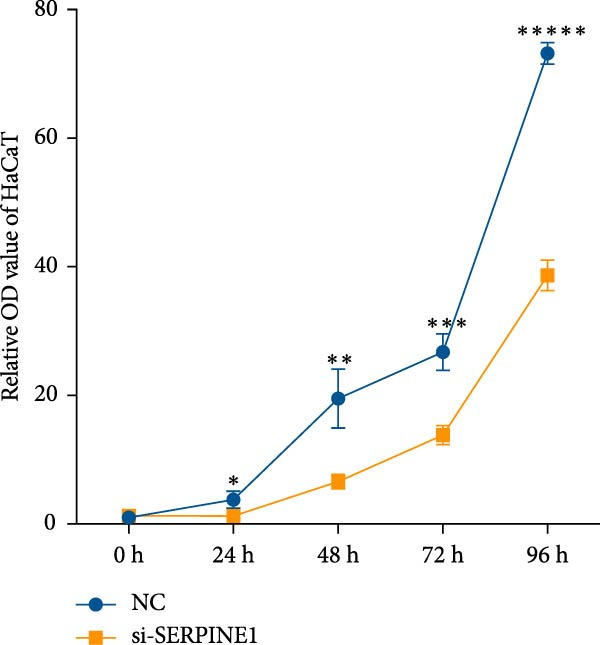
(B)

**Figure:**
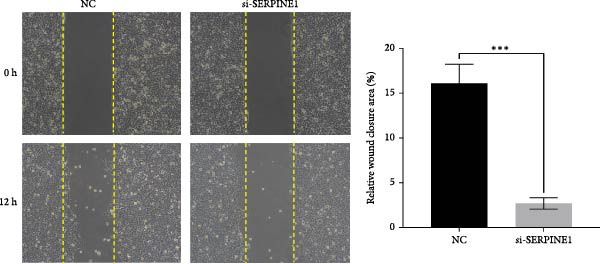
(C)

**Figure:**
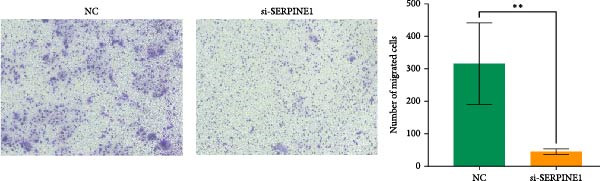
(D)

Upon specific knockdown of SERPINE1 expression in HDFs using siRNA, cell viability assessments with the CCK‐8 assay revealed that the proliferation rate of the si‐SERPINE1 group was significantly lower than the CON group on days 1, 4, and 5, indicating that SERPINE1 knockdown suppresses HDF proliferation (Figure 6B). In the cell scratch assay, the migration velocity of cells in the si‐SERPINE1 group was significantly slower than the CON group, suggesting that SERPINE1 knockdown reduces HDF migratory capacity (Figure 6C). Consistently, in the transwell assay, fewer HDFs from the si‐SERPINE1 group migrated through the inserts compared to the CON, further supporting that SERPINE1 knockdown decreases HDF migration (Figure 6D). Collectively, these experiments demonstrate that knocking down SERPINE1 in HDFs diminishes both their proliferative and migratory abilities.

Following the knockdown of SERPINE1 in HaCaT cells using siRNA, cell proliferation assessed by CCK‐8 assay showed that the proliferation rate of the si‐SERPINE1 group was significantly reduced compared to the CON group on days 4 and 5, implying that SERPINE1 suppression inhibits HaCaT proliferation (Figure 7B). In the scratch assay, the migration speed of cells in the si‐SERPINE1 group was significantly slower than the CON, indicating that SERPINE1 knockdown decreases HaCaT migratory potential (Figure 7C). Likewise, in the transwell assay, a significantly smaller number of HaCaT cells from the si‐SERPINE1 group migrated through the membrane compared to the CON, suggesting that SERPINE1 knockdown impairs HaCaT migration (Figure 7D). These collective findings indicate that SERPINE1 knockdown in HaCaT cells leads to a reduction in both proliferation and migration capabilities.

### 4.7. Phenotypic Validation by Overexpression of SERPINE1 in HDF and HaCaT Cells

Initially, we validated the efficiency of overexpression of SERPINE1 in HDF and HaCaT cells. The overexpression lentivirus significantly elevated SERPINE1 mRNA levels in both HDFs and HaCaT cells (Figures 8A and 9A), confirming successful augmentation of SERPINE1 expression in these cells.

Figure 8Phenotypic validation by overexpression of SERPINE1 in HDFs. (A) mRNA expression profiles in HDFs following lentiviral overexpression of SERPINE1. OE: SERPINE1 overexpressed by lentivirus; NC (OE): negative control group transduced with empty lentivirus; CON: untreated control group. (B) Overexpression of SERPINE1 facilitates proliferation in HDFs, measured using the CCK‐8 assay. (C) Cell scratch assay images displaying the dynamic change in oe‐SERPINE1 HDF migration capacity over 24 h (0–24 h) (200x magnification) (*n* = 3). (D) Transwell cell migration assay showed that overexpression of SERPINE1 promotes migration in HDFs. (200x magnification) (*n* = 3).  ^∗^ *p* < 0.05,  ^∗∗^ *p* < 0.01,  ^∗∗∗^ *p* < 0.001.

**Figure:**
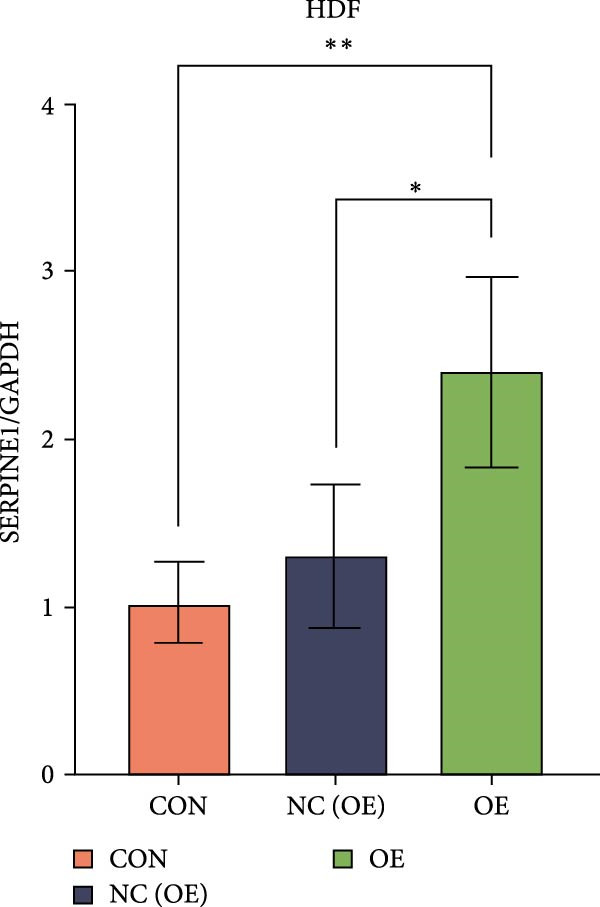
(A)

**Figure:**
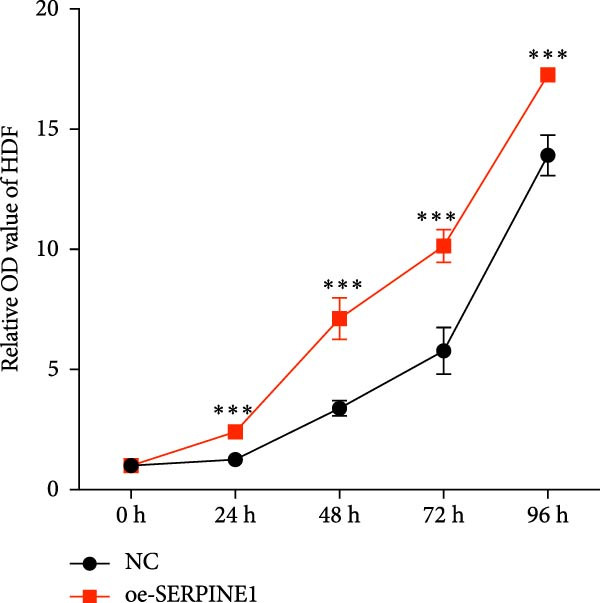
(B)

**Figure:**
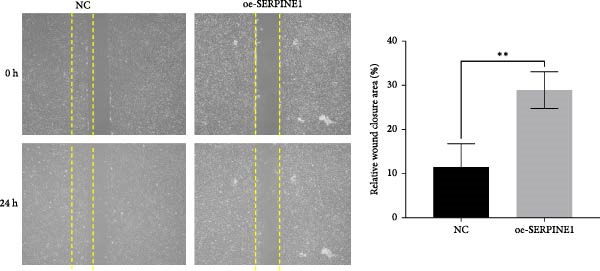
(C)

**Figure:**
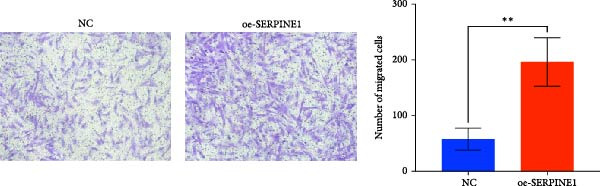
(D)

Figure 9Phenotypic Validation by Overexpression of SERPINE1 in HaCaT cells. (A) mRNA expression profiles in HaCaT following lentiviral overexpression of SERPINE1. OE: SERPINE1 overexpressed by lentivirus; NC (OE): negative control group transduced with empty lentivirus; CON: untreated control group. (B) Overexpression of SERPINE1 facilitates proliferation in HaCaT measured using the CCK‐8 assay. (C) Cell scratch assay images displaying the dynamic change in oe‐SERPINE1 HaCaT migration capacity over 24 h (0–24 h) (200x magnification) (*n* = 3). (D) Transwell cell migration assay showed that overexpression of SERPINE1 promotes migration in HaCaT. (200x magnification) (*n* = 3).  ^∗^ *p* < 0.05,  ^∗∗^ *p* < 0.01,  ^∗∗∗^ *p* < 0.001.

**Figure:**
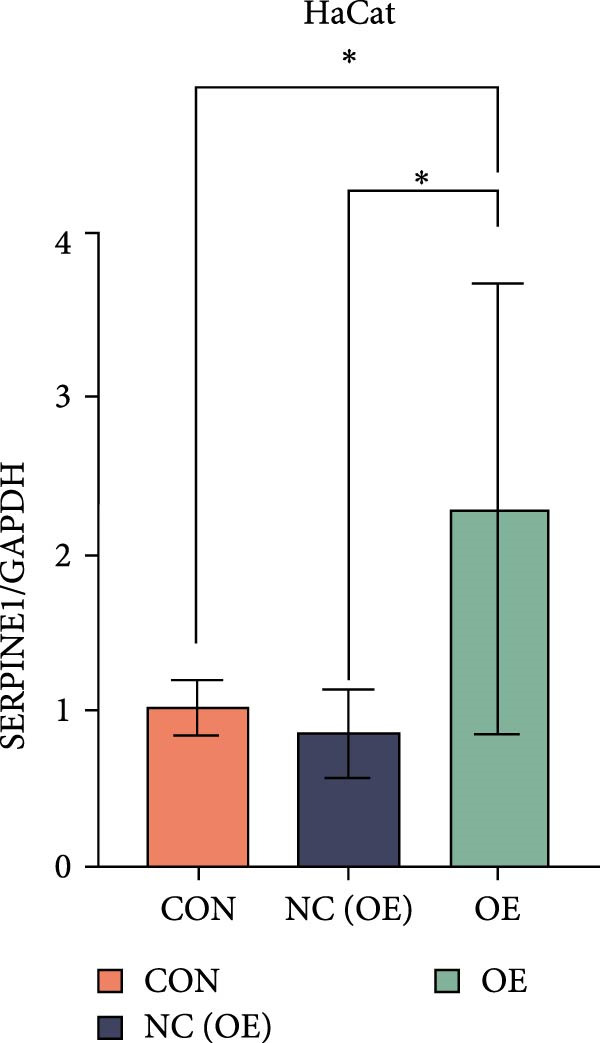
(A)

**Figure:**
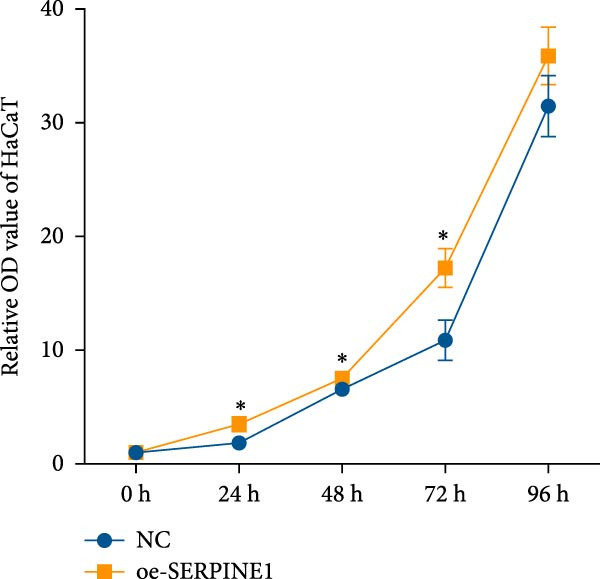
(B)

**Figure:**
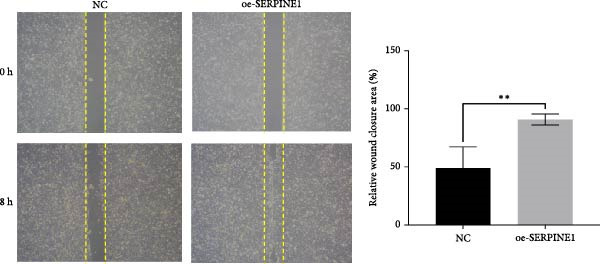
(C)

**Figure:**
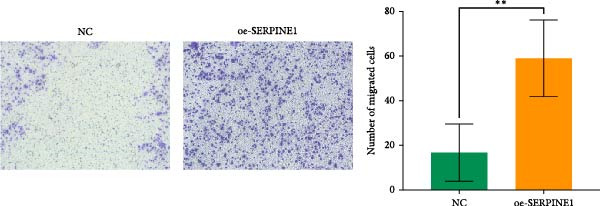
(D)

Upon overexpression of SERPINE1 in HDFs utilizing a lentiviral vector, cell viability measured by the CCK‐8 assay revealed that the OD values of the oe‐SERPINE1 group were significantly higher than the CON group on days 4, 5, and 6, suggesting that SERPINE1 overexpression enhances HDF proliferation (Figure 7C). In the transwell assay, a significantly greater number of HDFs from the oe‐SERPINE1 group migrated through the inserts compared to the CON, indicating that SERPINE1 overexpression elevates HDF migratory ability (Figure 7D). Collectively, these experiments illustrate that overexpressing SERPINE1 augments both the proliferative and migratory capacities of HDFs.

After overexpressing SERPINE1 in HaCaT cells using a lentiviral vector, cell viability assessment by CCK‐8 assay demonstrated that the OD values of the oe‐SERPINE1 group were significantly higher than the CON group on days 4 and 5, indicating that SERPINE1 overexpression enhances HaCaT proliferation (Figure 7E). In the scratch assay, the migration rate of the oe‐SERPINE1 group HaCaT cells was significantly faster than the CON, suggesting that SERPINE1 overexpression improves HaCaT migratory capacity (Figure 7F). Moreover, in the Transwell assay, a significantly larger number of HaCaT cells from the oe‐SERPINE1 group migrated through the inserts compared to the CON (Figure 7G), further implicating that SERPINE1 overexpression elevates HaCaT migration ability. Collectively, these experiments demonstrate that overexpressing SERPINE1 boosts both the proliferative and migratory capabilities of HaCaT cells.

### 4.8. Transcriptome Analysis Reveals the Differentially Expressed Genes and Pathways Influenced by SERPINE1

To further explore the downstream signaling pathways regulated by SERPINE1, transcriptome sequencing was performed after knocking down SERPINE1 in HDF. Using a cutoff of *padj* < 0.05 and |log2 fold change| > 1.0, we identified 725 upregulated genes and 672 downregulated genes (Figure 10A). The heat map showed the representative genes positively and negatively regulated by SERPINE1. The negatively regulated genes included *CAPN6*, *RSPO3*, *EGFL6*, *OGN*, *and LNX1*, and the positively regulated genes included *CSF2*, *ZNF24TR*, *CXCL8*, *KRTAP3-1*, and *CXCL1* (Figure 10B). To investigate the SERPINE1‐related signaling pathways, pathway enrichment analysis was performed. KEGG analysis of these upregulated and downregulated differentially expressed genes revealed that they were enriched in KEGG pathways, including PI3K‐Akt, MAPK, FoxO, Wnt, Hippo, Rap1, and Ras signaling pathways (Figure 10C). The enrichment of the PI3K‐Akt and MAPK pathways is of particular importance, as they are well‐established master regulators of cell survival, proliferation, and migration, processes that are central to tissue repair [21–23]. Concurrently, Gene Ontology (GO) analysis revealed significant enrichment in biological processes essential for wound healing, including “ameboidal‐type cell migration,” “epithelial cell proliferation,” “ECM organization,” and “regulation of cell adhesion” (Figure 10D). Therefore, the transcriptomic data strongly suggest that SERPINE1 orchestrates a prohealing program in skin cells by modulating these key pathways and biological processes, providing a mechanistic explanation for the enhanced cell migration and proliferation observed in our functional experiments.

Figure 10Transcriptome analysis of HDF reveals the differentially expressed genes and pathways influenced by SERPINE1. (A) Volcano map of differentially expressed genes identified by RNA‐seq of HDF‐siSERPINE1 and HDF. (B) Heat map showing differentially expressed genes in HDF regulated by SERPINE1. (C) Bubble diagram of KEGG enrichment analysis. (D) GO enrichment analysis histogram. GO, Gene Ontology; KEGG, Kyoto Encyclopedia of Genes and Genomes.

**Figure:**
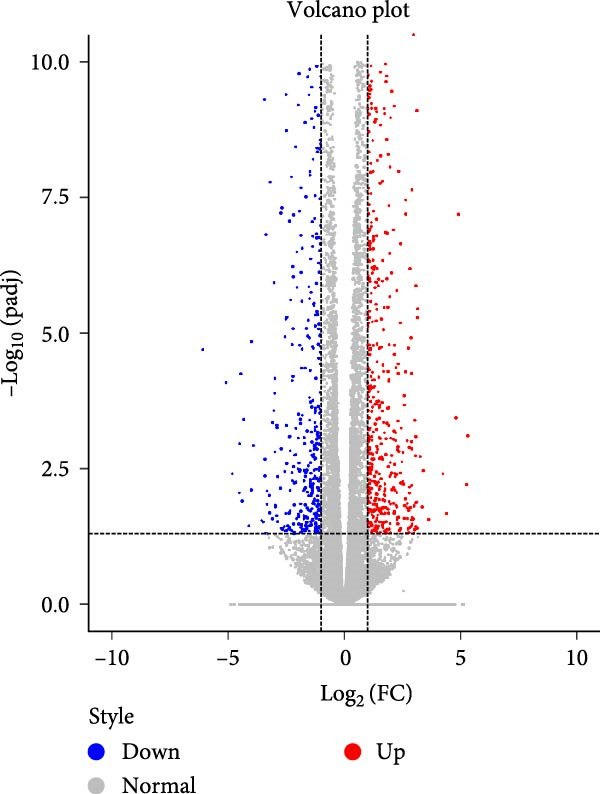
(A)

**Figure:**
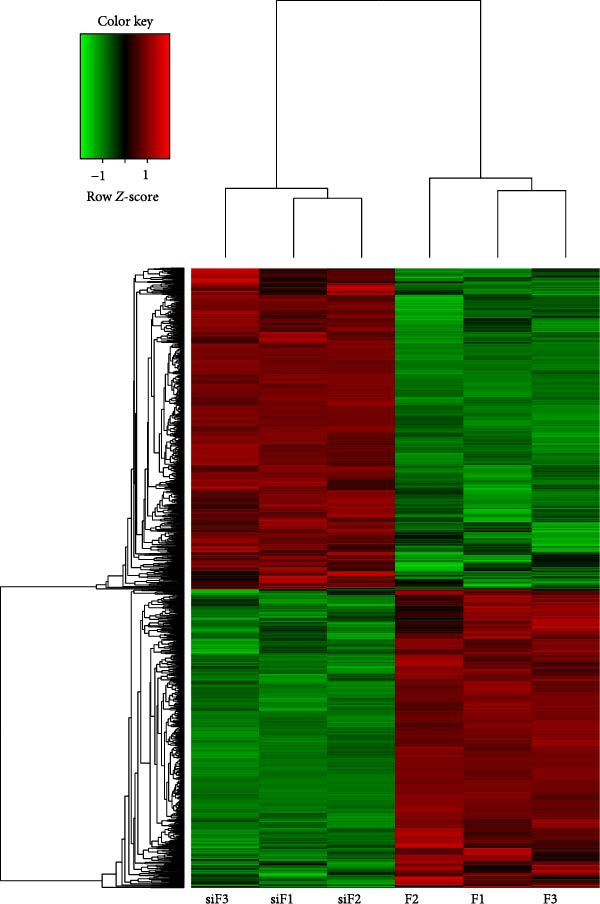
(B)

**Figure:**
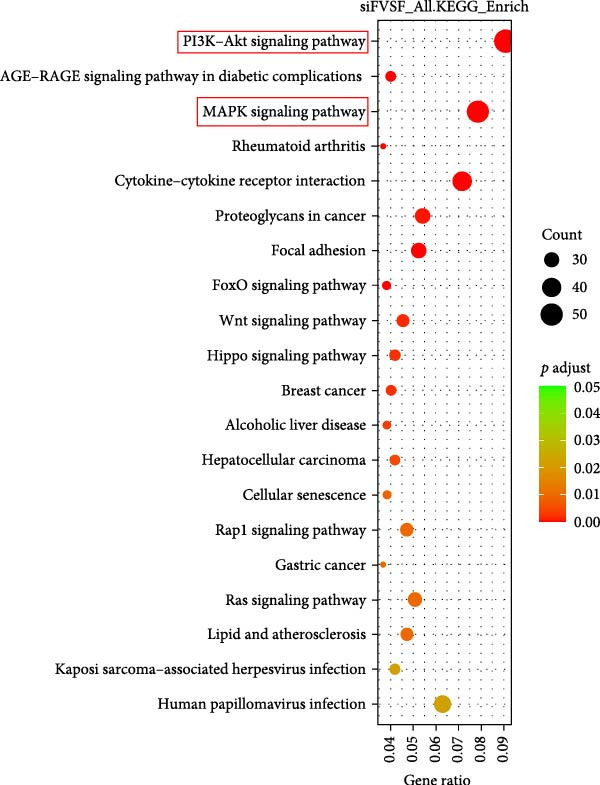
(C)

**Figure:**
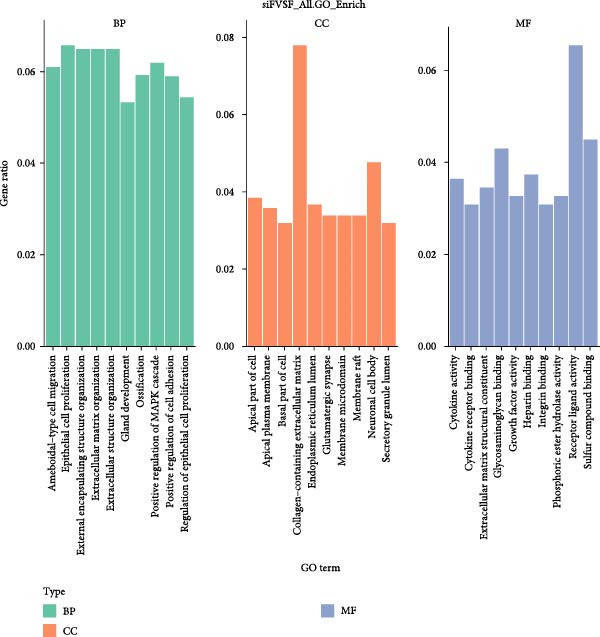
(D)

## 5. Discussion

Wound healing, a vital physiological process of natural tissue repair in response to injury, strictly adheres to successive stages including hemostasis, inflammation, proliferation, and remodeling. Delayed wound healing not only increases the risk of infection but may also lead to pain, discomfort, functional impairment, scarring, and heightened medical expenses. The adipose tissue, usually a medical waste after liposuction surgery, has been reported to contain a population of multipotent stem cells exhibiting characteristics of MSCs. In the present study, we report that hADSCs can be easily isolated from the donor’s sucked adipose without ethical concerns. Our data indicated that ADSCs can promote skin wound healing through dermal and epidermal cells and activate certain biological functions, such as proliferation and migration. This stimulatory effect of ADSCs on cutaneous wound healing may be partially mediated by the paracrine effects of ADSCs on other skin cells. By integrating high‐throughput sequencing, SERPINE1 was identified for the first time as a mediator in enhancing HDF and HaCaT proliferation and migration, which was further validated by the phenotypic studies after SERPINE1 knockdown and overexpression. What is more, pathway enrichment analysis showed that SERPINE1‐related signaling pathways included PI3K‐Akt, MAPK, FoxO, Wnt, Hippo, Rap1, and Ras signaling pathways. GO analysis identified ameboidal‐type cell migration, epithelial cell proliferation, external encapsulating structure organization, ECM organization, as well as regulation of cell adhesion as potential drivers. Overall, our results demonstrate that ADSCs enhance the functions of keratinocytes and fibroblasts by regulating SERPINE1 and represent a new approach to wound repair.

Adipose‐derived stem cells, with their defined multipotent capacity, abundant availability, minimal invasiveness, low adverse reactions, expandability, and immune tolerance, have emerged as one of the most promising alternatives for tissue regeneration, immune modulation, and facilitation of repair. ADSCs are known to proliferate and differentiate into skin cells to repair damaged or dead cells [1]. Accumulating evidence suggests that ADSCs can secrete a rich secretome involving cytokines, growth factors, and chemokines, which allow ADSCs to act as paracrine tools that are more likely than cell replacement [1, 4, 6]. The in vivo impact of ADSCs on wound repair was explored by a full‐thickness skin defect mouse model, verifying the efficacy of ADSCs in promoting wound healing. Histological analysis revealed that ADSCs reduced inflammation at the wound site, facilitated epithelialization, promoted collagen deposition, and accelerated neovascularization and myofibroblast formation, highlighting the potential clinical value of ADSCs in wound healing. Furthermore, we established a coculture system in vitro to evaluate the paracrine effect of ADSCs on the key cell types in the wound healing process, HDFs and HaCaT. Upon coculture with HDFs and HaCaT cells, ADSCs were shown to enhance their proliferation and migration. This observation is important because cell migration and proliferation are essential steps during wound healing [24].

To identify the critical molecule, we conducted transcriptome sequencing of cocultured HDFs and HaCaT cells, with SERPINE1 being pinpointed. As the major physiologic regulator of the pericellular plasmin‐generating cascade [25, 26], SERPINE1 was found to modulate cellular adhesion or migration, wound healing, angiogenesis, and tumor cell metastasis [27, 28]. Previous studies by Sun HJ et al. revealed that SERPINE1 played a role in mediating epithelial wound healing and its impaired expression may contribute to delayed wound healing in DM corneas [29]. In the process of wound healing, SERPINE1 was activated early after wounding, and varied functions suggest a key role in the repair transcriptome and cutaneous injury response program[12]. It was reported that hAMSC‐CM contained high levels of PAI‐1 [30], and MSC‐derived PAI‐1 significantly increased fibroblast migration in vitro and improved wound healing in vivo by decreasing time to wound closure [31]. To date, the role of SERPINE1 and/or the uPA proteolytic pathway in wound healing in normal conditions and delayed wound repair remains elusive. Therefore, we followed up on our RNA sequencing results and further solidified the pivotal role of SERPINE1 in modulating the HDFs and HaCaT cells through gene knockdown and overexpression approaches. We found that inhibition of SERPINE1 impaired the proliferation and migration of HDFs and HaCaT cells, whereas oe‐SERPINE1 accelerated the proliferation and migration of HDFs and HaCaT cells. These results suggested ADSC may target SERPINE1 gene expression and/or SERPINE1 function by paracrine effect and then promote the wound healing process.

SERPINE1/PAI‐1 regulates proliferation, apoptosis, angiogenesis, invasion, metastasis, and inflammation in different cell lines by activating the ERK, AKT, JAK–STAT, and NF‐κB signaling pathways in cancer and fibrotic disease [32, 33]. Our transcriptome analysis upon SERPINE1 knockdown in HDFs confirmed that it significantly modulates key signaling pathways, with the PI3K‐Akt and MAPK pathways emerging as the most prominently enriched hubs. This positions them as the core downstream machinery for SERPINE1 in our model, effectively bridging the gap between ADSC paracrine signaling and intracellular activation. Substantiating this link, the PI3K/AKT pathway is a well‐documented target of PAI‐1 in wound healing contexts [34], and its activation by stem cells, as shown by Li JY et al. [30], aligns perfectly with our findings. Similarly, the MAPK/ERK pathway, a critical driver of proliferation [22], is not only an inducer of PAI‐1 [35] but also a key output of it, as per our data, suggesting a potent reinforcing signaling loop. Crucially, the corresponding GO analysis confirmed that this pathway activation directly translates into the execution of essential healing processes, including epithelial cell proliferation, ECM organization, and ameboidal‐type cell migration. Collectively, our results substantiate a model wherein ADSCs augment the migration and growth capabilities of fibroblasts and keratocytes by SERPINE1 in the context of cutaneous wound healing.

It has been well‐documented that the paracrine factors, such as growth factors, cytokines, and exosomes of stem cells, contribute to the therapeutic effect [36–38]. Our experimental data, derived from a Transwell coculture system that prevents direct cell contact, provide preliminary evidence consistent with a paracrine mechanism in the ADSC‐mediated upregulation of SERPINE1 in HDFs and HaCaT cells. SERPINE1 expression is known to be regulated by multiple modulators, including growth factors and cytokines. For instance, its expression has long been recognized as TGFβ1‐mediated by binding to uPA and uPAR and activating MAPK [29], and Sun et al. reported that MSC paracrine factors could induce macrophage secretion of PAI‐1[39]. Furthermore, a broad spectrum of cytokines, such as uPAR, IL‐6, OPN, ANG‐2, HGF, TGFb1, RBP4, ANG‐1, FAP, IL‐11, Follistatin, and DcR3, capable of activating the PI3K/AKT pathways, are secreted by hAMSCs [30]. However, in the present study, we didn’t find such a cytokine as we thought these cytokines do not operate in isolation but rather interact with other regulatory proteins. No single growth factor could reproduce the activity of ADSCs, and a combination of several growth factors, ECM proteins, or other unknown substances might account for the full effect of ADSCs [40]. What is more, the specific identity of the key ADSC‐derived paracrine factors responsible for SERPINE1 induction in our model remains to be definitively elucidated. Therefore, while our data support the involvement of a paracrine mechanism, future work is necessary to dissect this complex secretome. A primary focus will be to determine the specific contribution of exosomes, utilizing inhibition approaches (e.g., GW4869), coupled with detailed cargo profiling (e.g., miRNA sequencing) and functional rescue experiments, to precisely delineate the upstream regulators of SERPINE1 in this context.

In summary, this study explored the potential application of ADSCs in a therapeutic approach to skin wound healing. We uncovered that ADSCs can enhance the proliferation and migration functions of two major skin cells by regulating SERPINE1, and then can promote the process of wound healing. These findings enrich our understanding of the mechanisms underlying ADSCs’ role in tissue repair and regeneration, offering valuable insights for clinical applications.

## 6. Conclusion

In this research, we successfully extracted and characterized ADSCs. The in vivo model and in vitro experiments demonstrated that ADSC effectively promotes cutaneous wound healing by augmenting the proliferation and migration of fibroblasts and keratinocytes through upregulating SERPINE1. RNA sequencing analysis revealed that the potential downstream of SERPINE1 are mainly related to PI3K‐Akt and MAPK signaling pathways. These findings not only augment our understanding of the mechanisms underlying ADSCs’ actions in tissue repair and regeneration but also furnish invaluable insights for clinical applications. Specifically, they pave the way for the development of targeted therapeutic drugs or innovative biomaterials designed for wound healing, thereby offering new theoretical foundations for clinical practice. This research fosters the conception of more efficacious and precise interventions in wound management and therapy, steering the field toward advanced strategies in treating wounds.

## Ethics Statement

This study was performed according to the principles of the Declaration of Helsinki. The human adipose samples were collected following informed consent. The use of human adipose tissue was approved by the Clinical Research Ethics Committee of the First Affiliated Hospital, College of Medicine, Zhejiang University (Project title: Study on the mechanism of Adipose Mesenchymal Stem Cells participating in skin wound healing; Approval date: 2020.05.25; Approval number: 2020IIT943). Animal experiments in this study were approved by the Tab of Animal Experimental Ethical Inspection of the First Affiliated Hospital, College of Medicine, Zhejiang University (Project title: Study on the mechanism of Adipose Mesenchymal Stem Cells participating in skin wound healing; Approval date: 2020.05.25; Approval number: 2020–1294).

## Disclosure

All authors confirm their consent for publication.

## Conflicts of Interest

The authors declare no conflicts of interest.

## Author Contributions

Conceptualization, writing – review and editing: Yijia Yu and Jinghong Xu. Methodology: Jiaqi Sun, Mingyuan Xu, YeHua Liang, and Qinqian Sun. Experiments: YeHua Liang, Qinqian Sun, and Jiaqi Sun. Data analysis, writing – original draft: YeHua Liang and Qinqian Sun.

## Funding

This work was supported by the Zhejiang Provincial Natural Science Foundation of China (Grant LQ21H150005).

## Data Availability

The datasets used and/or analyzed during the current study are available from the corresponding author upon reasonable request.
